# Epigenetic Regulation of HIV-1 Latency by Cytosine Methylation

**DOI:** 10.1371/journal.ppat.1000495

**Published:** 2009-06-26

**Authors:** Steven E. Kauder, Alberto Bosque, Annica Lindqvist, Vicente Planelles, Eric Verdin

**Affiliations:** 1 Gladstone Institute of Virology and Immunology, San Francisco, California, United States of America; 2 Department of Medicine, University of California, San Francisco, California, United States of America; 3 Department of Pathology, University of Utah, Salt Lake City, Utah, United States of America; 4 Department of Laboratory Medicine, Division of Clinical Microbiology, Karolinska Institutet, Karolinska University Hospital, Stockholm, Sweden; University of Pennsylvania School of Medicine, United States of America

## Abstract

Human immunodeficiency virus type 1 (HIV-1) persists in a latent state within resting CD4^+^ T cells of infected persons treated with highly active antiretroviral therapy (HAART). This reservoir must be eliminated for the clearance of infection. Using a cDNA library screen, we have identified methyl-CpG binding domain protein 2 (MBD2) as a regulator of HIV-1 latency. Two CpG islands flank the HIV-1 transcription start site and are methylated in latently infected Jurkat cells and primary CD4^+^ T cells. MBD2 and histone deacetylase 2 (HDAC2) are found at one of these CpG islands during latency. Inhibition of cytosine methylation with 5-aza-2′deoxycytidine (aza-CdR) abrogates recruitment of MBD2 and HDAC2. Furthermore, aza-CdR potently synergizes with the NF-κB activators prostratin or TNF-α to reactivate latent HIV-1. These observations confirm that cytosine methylation and MBD2 are epigenetic regulators of HIV-1 latency. Clearance of HIV-1 from infected persons may be enhanced by inclusion of DNA methylation inhibitors, such as aza-CdR, and NF-κB activators into current antiviral therapies.

## Author Summary

Current drug therapies inhibit replication of the human immunodeficiency virus (HIV). In patients undergoing these therapies, the amount of HIV is reduced to an undetectable level and HIV-related disease subsides. However, stopping antiviral drug therapy results in the quick return of HIV and of disease. One reason for this is latently infected cells, in which virus replication is temporarily halted. When drug therapy is stopped, virus from these latently infected cells can resume infection and spread to other cells in the patient, resulting in the return of disease. Here, we demonstrate that one mechanism of latency is DNA methylation, in which chemical groups called methyl groups are added to HIV DNA. We also identify a host protein called methyl-CpG binding domain protein 2 (MBD2) that binds methylated HIV DNA and is an important mediator of latency. Furthermore, we demonstrate that a drug that inhibits DNA methylation potently reactivates latent HIV. Novel strategies to eliminate or reduce the latent reservoir are necessary. Our findings may prove useful in the development of novel therapies to efficiently reactivate latent HIV-1, thus making it susceptible to current drug therapies.

## Introduction

In HIV-infected individuals, highly active anti-retroviral therapy (HAART) dramatically reduces HIV-1 plasma titers [1]–[3] and decreases morbidity and mortality [4]. However, a reservoir of latent virus persists within resting CD4^+^ T cells [5]–[8] and contributes to the reemergence of viremia upon discontinuation of HAART [9]–[11]. Reactivation of latent HIV-1, thus rendering it susceptible to HAART, is a critical component of any strategy for HIV-1 clearance [12]–[14]. Transcriptional repression is an important component of HIV-1 latency, necessitating identification of cellular proteins that repress HIV-1 transcription and the testing of small molecules that inhibit these cellular proteins.

In resting CD4^+^ T cells, HIV-1 is maintained in a latent state by multiple factors that inhibit virus gene expression after integration into cellular DNA. In particular, several studies have highlighted the critical role of chromatin structure at the site of provirus integration in repressing provirus transcription. Sequence-specific transcription factors can recruit histone deacetylases (HDACs) and other chromatin-modifying enzymes to the provirus promoter, resulting in transcriptional repression and virus latency [15]–[19]. Interestingly, the mechanism by which virus escapes silencing by these sequence-specific factors in a productive infection is unknown. Additionally, resting CD4^+^ T cells are deficient in transcription factors essential for HIV-1 transcription [20], and latent virus can be reactivated by stimulation of T cell pathways that activate these factors [5]–[8]. The provirus integration site can also be a determinant of latency, either by making the provirus susceptible to transcriptional interference from cellular genes [21]–[24] or by suppressing virus transcription through the formation of heterochromatin [25]. Post-transcriptional mechanisms affecting the export [26] or translation [27] of HIV-1 mRNAs constitute other blocks to HIV-1 gene expression during latency.

The resting state of CD4^+^ T cells and the activity of HDACs are two of the best-understood characteristics of latency, but stimulation of resting CD4^+^ T cells or inhibition of HDACs in HIV-infected patients do not appreciably decrease the latent reservoir when combined with HAART [28]–[32].

The study of latently infected cells is hampered by their rarity in HIV-infected individuals and the lack of a marker for latent infection. For these reasons, we developed the J-Lat cell lines as an *in vitro* model of HIV-1 latency [33]. Similar to latently infected CD4^+^ T cells, the J-Lat cells harbor a full-length HIV-1 genome that is transcriptionally competent, is integrated within actively transcribed cellular genes, and is inhibited at the transcriptional level. Additionally, the latent provirus integrated in the J-Lat cell lines encodes the GFP gene, providing a fluorescent marker of HIV-1 transcriptional activity.

To identify novel mechanisms of HIV-1 latency, we have conducted a cDNA screen in J-Lat cells for genes that reactivate latent HIV-1. This screen identified a portion of methyl-CpG binding domain protein 2 (MBD2), a transcriptional repressor that binds methylated DNA. We found that the HIV-1 promoter is hypermethylated in J-Lat cell lines and in primary CD4 T cells at two CpG islands surrounding the HIV-1 transcriptional start site. Most importantly, we found that a small molecule inhibitor of DNA methylation, 5-aza-2′deoxycytidine (aza-CdR), synergizes with NF-κB activators to promote a dramatic increase in virus gene expression. Aza-CdR is approved for use in humans to treat myelodysplastic syndrome [34] and may promote the reactivation of latent HIV-1 and the clearance of latently-infected cells in combination with HAART in HIV-infected patients.

## Results

### A genetic screen to identify novel regulators of HIV-1 latency

**Figure 1:**
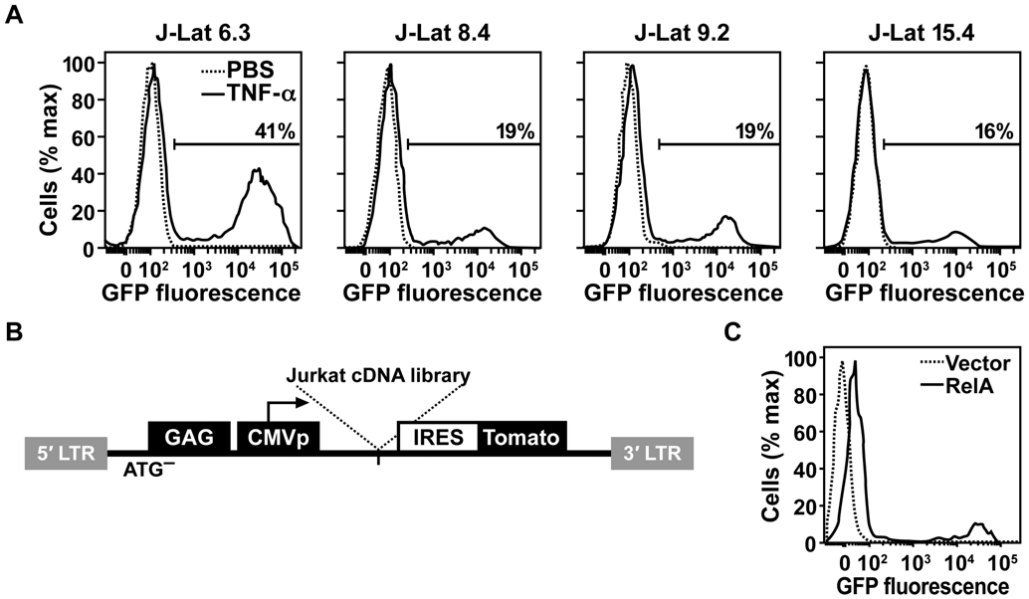
A cDNA screen to identify novel regulators of HIV-1 latency. (A) Flow cytometry of latent HIV-1 reactivation in indicated J-Lat cell lines after treatment with PBS or TNF-α. GFP-positive cells are indicated by gate and percentage. (B) Genome of retrovirus vector used in expression screen. (C) Flow cytometry of latent HIV-1 reactivation in J-Lat 6.3 cells after transduction with retrovirus expression vectors marked with the tomato fluorescent protein. Histograms indicate GFP expression after gating for tomato-positive cells.

The J-Lat cells are clonal cell lines isolated after infection of Jurkat cells with a HIV-1 virus encoding GFP. Latently infected cells were selected that were GFP-negative at the basal state but became GFP-positive after treatment with TNF-α. Treatment of each cell line with TNF-α reactivated latent HIV-1 to a different extent, depending on the cell line (Figure 1A). To identify cellular genes that control HIV-1 latency in this system, a complementary DNA (cDNA) library was made from the Jurkat T cell line and cloned into a plasmid encoding the pBMN-CSI-T retrovirus vector, which expresses tomato fluorescent protein as a marker (Figure 1B).

To confirm that this vector mediates expression of cloned cDNAs at a level sufficient for reactivation of latent HIV-1, a positive control virus was produced that encodes NF-κB RelA, which reactivates latent HIV-1 in J-Lat cells [18]. Infection of J-Lat cell line 6.3 with the RelA-encoding virus caused a 3.5-fold increase in HIV-1 gene expression compared to a control virus that lacks an insert (Figure 1C).

The cDNA library was packaged into retroviral particles and introduced into the J-Lat 6.3 cell line via infection (Table 1). GFP-positive cells, indicative of reactivated latent HIV-1, were isolated by fluorescence activated cell sorting (FACS). cDNA library inserts were amplified from genomic DNA obtained from these cells by PCR with virus-specific primers (Figure S1A) and recloned into pBMN-CSI-T.

**Table 1:** cDNA screening.

Library complexity	1,000,000 independent clones
Cells analyzed	15,000,000
Cells receiving cDNA insert (live gate)^a^	2,100,000 (14%)
Cells with reactivated HIV-1 (live gate)^b^	58,235
Cells with cDNA insert and reactivated HIV-1^c^	11,122 (19%)
cDNA clones^d^	11,122

a Tomato-positive cells in live gate. Percentage is consistent with single-hit infection kinetics.

b GFP-positive cells in live gate.

c Tomato-positive cells in the GFP-positive gate. Greater number of tomato-positive cells than in live gate, indicating that GFP selection enriches for cDNAs that reactivate latent HIV-1.

d Clones that potentially reactivate latent HIV-1.

### MBD2 regulates HIV-1 latency and repression of methylated DNA

One clone identified in this screen, MBD2_1345-1947_, corresponded to nucleotides 1345–1947 of the mRNA encoding the MBD2 transcriptional repressor (Figure S2). Importantly, the first ATG within this clone is in frame with the authentic MBD2 initiation codon, indicating a truncated protein corresponding to amino acids 388 to 411 of full-length MBD2 could be translated.

MBD2 is a member of the methyl-CpG binding domain family of proteins, which possess methyl-CpG binding domains (MBDs). Similar to other members of this family, MBD2 specifically binds methylated DNA and mediates transcriptional repression by recruitment of the nucleosome remodeling and histone deacetylation (NuRD) complex that includes chromatin remodeling and HDAC activities [35]–[37].

**Figure 2:**
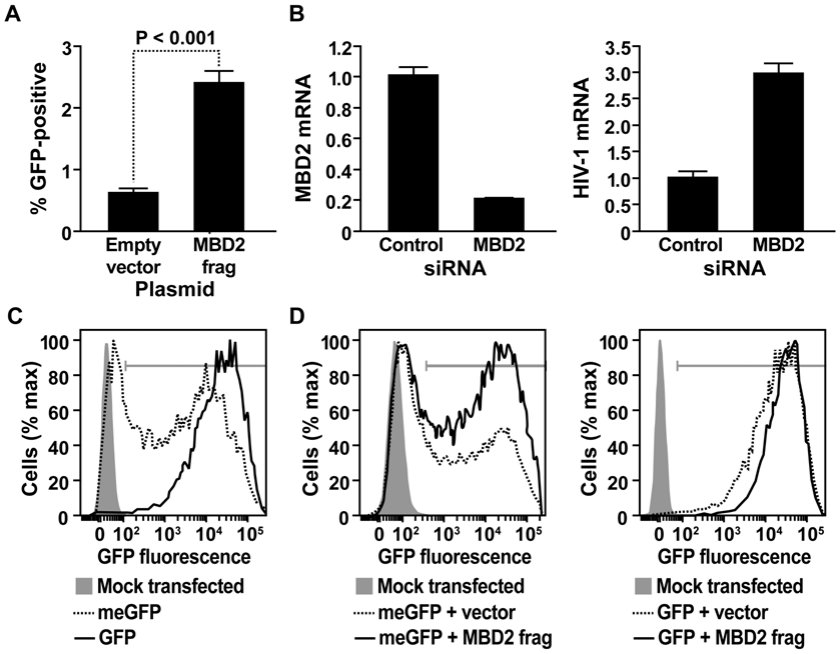
MBD2 regulates HIV-1 latency and transcriptional repression. (A) Latent HIV-1 reactivation in J-Lat 6.3 cells after transfection with the indicated expression plasmids and flow cytometry. Percent GFP-positive cells after gating for tomato-positive cells is shown. Experiment was performed in triplicate and error bars represent standard deviation. (B) Transcriptional activation of latent HIV-1 in J-Lat 6.3 after transfection with a siRNA that targets MBD2. Levels of MBD2 (left panel) or HIV-1 (right panel) mRNA were determined by reverse transcription and quantitative PCR and normalized to those after transfection with a non-targeting control siRNA. Data are representative of three different experiments. Error bars indicate standard deviation of qPCR results. (C) Flow cytometry of GFP expression in 293T cells that were mock transfected, transfected with methylated pEGFP-N1 (meGFP), or with unmethylated pEGFP-N1 (GFP). Gate indicates GFP-positive cells. (D) Flow cytometry of GFP expression in 293T cells cotransfected with methylated pEGFP-N1 (left panel) or unmethylated pEGFP-N1 (right panel) and an expression vector marked by the tomato fluorescent protein. Tomato-positive cells were gated to measure GFP expression in populations that received a control vector lacking an insert or one that encodes MBD2_1345–1947_. Gates indicate GFP-positive cells.

To confirm that MBD2_1345–1947_ reactivates latent HIV-1, J-Lat cells were transfected with an expression vector for this polypeptide. Transfection of J-Lat 6.3 with MBD2_1345–1947_ induced a 5-fold greater reactivation of latent HIV-1 in comparison to an empty vector control (Figure 2A). Since MBD2 inhibits transcription of methylated DNA [35], the identification of a C-terminal fragment of MBD2 in our screen indicated that this fragment inhibits endogenous MBD2 function in a dominant-negative manner. Furthermore, identification of this fragment implicated full-length, endogenous MBD2 in the repression of HIV-1 transcription during latency. To establish the role of endogenous MBD2 in HIV-1 latency, J-Lat 6.3 was transfected with a pool of siRNAs corresponding to this factor. This resulted in an 80 percent reduction in the level of MBD2 mRNA compared to cells transfected with a non-targeting control siRNA pool (Figure 2B, left panel). Depletion of MBD2 resulted in a 300 percent increase in HIV-1 mRNA compared to those transfected with the control siRNA pool (Figure 2B, right panel). These data demonstrate that MBD2 participates in the repression of HIV-1 transcription during latency.

Since MBD2 inhibits transcription of methylated DNA [35], we believed the C-terminal MBD2 fragment identified in our screen might reactivate latent virus by inhibiting endogenous MBD2 function. To test MBD2_1345-1947_ for this activity, we examined its effect on transcription of methylated DNA in a heterologous system. 293T cells were cotransfected with an expression vector for MBD2_1345–1947_ and with another plasmid encoding GFP under the control of the CMV promoter (pEGFP-N1). This latter plasmid was either methylated *in vitro* (meGFP) or left unmethylated (GFP). Plasmid methylation was confirmed by resistance to *Hpa* II cleavage (Figure S1B) and reduced GFP expression in transfected 293T cells (Figure 2C). Importantly, cotransfection of the MBD2_1345–1947_ plasmid with methylated pEGFP-N1 increased the proportion of GFP-positive cells from 58 to 72 percent (Figure 2D, left panel). Furthermore, derepression by MBD2_1345–1947_ was preferential for methylated DNA, and a similar effect was not observed with non-methylated pEGFP-N1 (Figure 2D, right panel). These results implicate MBD2 and cytosine methylation in the regulation of HIV-1 latency in the J-Lat system.

### HIV-1 latency is associated with cytosine methylation in provirus CpG islands

**Figure 3:**
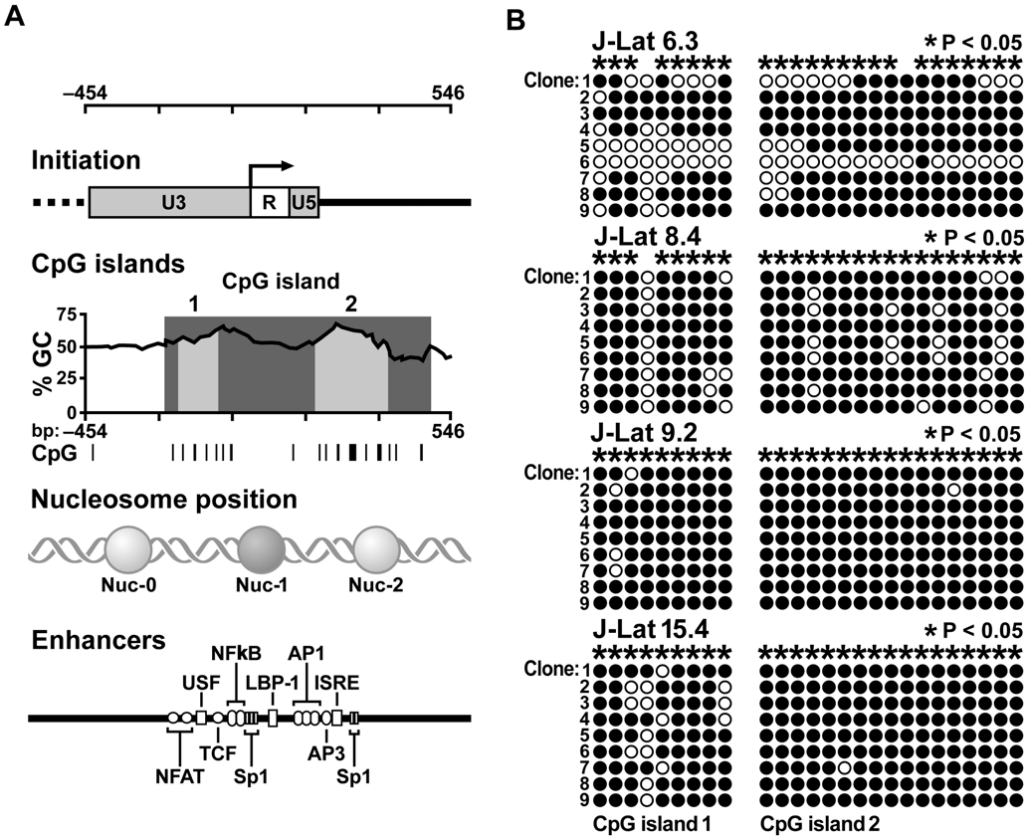
HIV-1 encodes two CpG islands that are methylated during latency. (A) Attributes of DNA surrounding HIV-1 transcript initiation site. Initiation: Transcription initiation site within HIV-1 LTR is indicated by arrow. CpG islands: Plot of GC content of DNA surrounding initiation site. Light grey areas indicate CpG islands 1 and 2. Vertical lines indicate CpGs. Nucleosome position: Locations of nucleosomes in HIV-1 promoter. Enhancers: Transcription factor binding sites. (B) Bisulfite-mediated methylcytosine mapping of latent HIV-1 in J-Lat cells. CpG islands 1 and 2 and J-Lat line are indicated. Each column represents one CpG position, with each circle in the column indicating either cytosine (open circles) or methylcytosine (filled circles) in an independently cloned DNA molecule. Asterisks indicate cytosines with statistically significant methylation.

Cytosine methylation is an epigenetic modification that inhibits transcription when CpG islands, clusters of CpG dinucleotides proximal to a transcription start site, are hypermethylated [38]. To determine whether the HIV-1 genome encodes CpG islands, the methprimer program [39] was used search the HIV-1 provirus nucleotide sequence. Two CpG islands were identified flanking the transcription start site at positions -194 to -94 and 180 to 368 (Figure 3A). These islands overlap with two regions that were previously shown to be nucleosome-free [40] and rich in transcription factor binding sites [41], two features usually associated with *bona fide* CpG islands [38]. To determine whether HIV-1 CpG islands are methylated during latency, their methylation state was analyzed by bisulfite-mediated methylcytosine mapping. Figure S3 shows nucleotide sequence of the HIV-1 promoter, positions of CpG islands, and the particular CpGs subjected to methylation analysis. We found that both CpG islands were hypermethylated in four different J-Lat cell lines, with the majority of CpGs methylated more than 70 percent of the time (Figure 3B and Figure S4A–D). In sodium bisulfite-treated DNA, cytosine was converted to thymine in greater than 99 percent of all CpN dinucleotides (N = A, T, or C), confirming efficient bisulfite conversion of non-methylated cytosines (Figure S5A).

### Cytosine methylation recruits transcriptional repressors to the HIV-1 promoter

**Figure 4:**
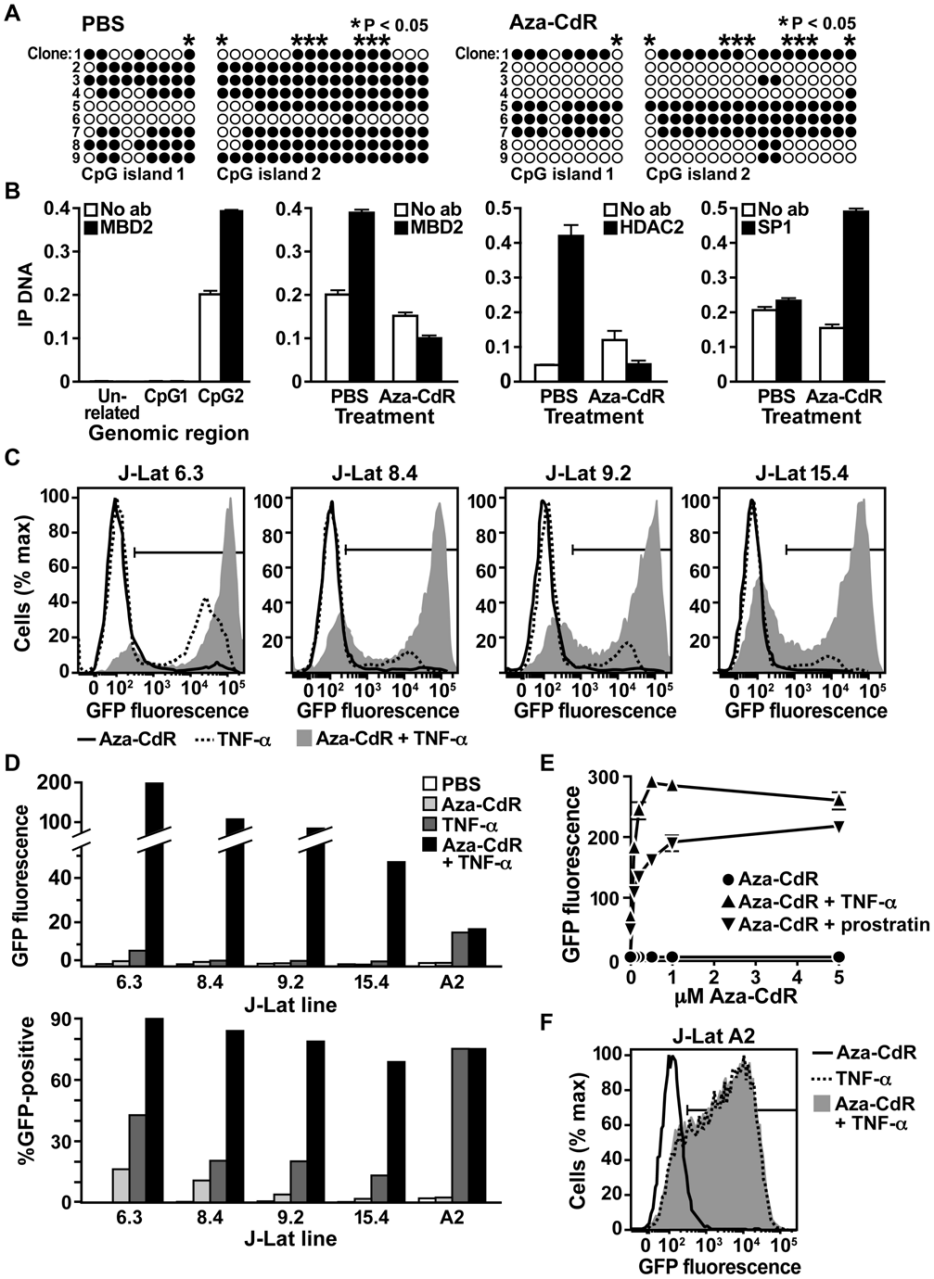
Methylation-dependent recruitment of transcription factors and maintenance of HIV-1 latency. (A) Bisulfite-mediated methylcytosine mapping of HIV-1 in J-Lat 6.3 cells treated with PBS (left panel) or aza-CdR (right panel). Asterisks indicate cytosines with a statistically significant lesser level of methylation in cells treated with aza-CdR. (B) Panel 1: Chromatin immunoprecipitation analysis of MBD2 recruitment to an unrelated DNA sequence between the β-actin and fascin-1 genes (unrelated), HIV-1 CpG island 1 (CpG1), and HIV-1 CpG island 2 (CpG2). Panels 2–4: Chromatin immunoprecipitation analysis of MBD2, HDAC2, or Sp1 recruitment to CpG island 2 in cells treated with PBS or aza-CdR. Data are representative of three independent experiments. Error bars indicate standard deviation of quantitative PCR results. (C) Flow cytometric analysis of latent HIV-1 reactivation in the indicated J-Lat clones after treatment with aza-CdR, TNF-α, or aza-CdR plus TNF-α. Histograms indicate GFP fluorescence. Gates indicate GFP-positive cells. (D) Bar graph representation of reactivation data. HIV-1 expression was normalized to control cells treated with PBS, and either GFP fluorescence (top panel) or percentage of GFP-positive cells (bottom panel) is displayed. Results are representative of three independent experiments. (E) Latent HIV-1 reactivation in J-Lat 6.3 treated with increasing doses of aza-CdR alone, in combination with TNF-α, or in combination with prostratin. Data points on the y-axis represent fluorescence at 0 uM Aza-CdR. GFP fluorescence was measured by flow cytometry and normalized to control cells treated with PBS. Error bars indicate standard deviation of three experiments. (F) Latent HIV-1 reactivation in the J-Lat cell line A2 treated with aza-CdR, TNF-α, or aza-CdR plus TNF-α. Gate indicates GFP-positive cells.

MBD2 mediates transcriptional repression by acting as a bridge between hypermethylated CpG islands and chromatin modifying enzymes, including HDACs [42]. To test whether MBD2 is recruited to the HIV-1 provirus *in vivo*, we performed chromatin immunoprecipitation (ChIP) assays. Chromatin from J-lat cells was incubated with MBD2 antisera and the immunoprecipitated material analyzed by quantitative PCR for presence of HIV-1 provirus. We observed recruitment of MBD2 to CpG island 2 of the HIV-1 genome, but observed no recruitment to CpG island 1 in comparison to a negative control (Figure 4B, first panel). Treatment of J-Lat 6.3 with aza-CdR, an inhibitor of DNA methylation, caused up to a 50 percent decrease in methylation, depending on the CpG analyzed (Figure 4A and Figure S4E), demonstrating that HIV-1 DNA methylation is reversible. It should be noted that the data for PBS-treated J-Lat 6.3 in Figures 3B and 4A are from the same experiment. Importantly, MBD2 recruitment to CpG island 2 was eliminated when cytosine methylation was inhibited by treatment of the cells with aza-CdR (Figure 4B, second panel). Next, we tested for the presence of HDAC2, an MBD2 cofactor, at CpG island 2 during latency. Comparable to MBD2, HDAC2 was recruited to CpG island 2 during latency and was lost after treatment with aza-CdR (Figure 4B, third panel). In contrast, inhibition of methylation by aza-CdR was associated with increased Sp1 recruitment to CpG island 2 (Figure 4B, fourth panel). These data demonstrate that during latency, multiple components of the NuRD complex recognize the methylated HIV-1 CpG island 2 and that this recruitment may be pharmacologically reversed.

### Synergistic reactivation of latent HIV-1 by aza-CdR and NF-κB activators

The finding that methylation of CpG islands flanking the HIV-1 transcription start site can be reversed with aza-CdR suggests that aza-CdR could reactivate latent HIV-1. Aza-CdR alone, however, showed little effect in terms of mean fluorescence intensity or the proportion of GFP-positive cells (Figures 4C and 4D). As previously observed, treatment with TNF-α reactivated latent HIV-1 in only a fraction of the cell population ranging from 16 to 41 percent depending on the J-Lat cell line studied (Figure 1A). In contrast, dual treatment of latently infected J-Lat clonal cell lines (lines 6.3, 8.4, 9.2 and 15.4) with both aza-CdR and TNF-α induced a dramatic increase in HIV-1 gene expression (Figures 4C and 4D). Powerful synergy was observed when aza-CdR was used at concentrations as low as 0.5 µM in combination with TNF-α or the NF-κB activator prostratin (Figure 4E). The combination of aza-CdR and TNF-α increased HIV-1 expression 196-, 101-, 76-, and 47-fold over PBS-treated control cells (Figure 4D, upper panel and Table S1). Each of these increases was nearly 20-fold greater than the additive effect of the two reagents (Table S1). Synergistic activation of transcription was specific for the HIV-1 promoter. Analysis of J-Lat 6.3 by RT-PCR found that aza-CdR and TNF-α synergistically activated HIV-1 transcription (Figure S6A, left panel) but had only an additive effect on transcription of IκB-α, another NF-κB-regulated gene (Figure S6A, right panel). The same result was observed for J-Lat 8.4. Aza-CdR and TNF-α synergistically activated HIV-1 transcription (Figure S6B, left panel) but had only an additive effect on transcription of IκB-α (Figure S6B, right panel). To determine if aza-CdR acts directly on HIV-1 transcription, J-Lat cells were treated with cycloheximide to inhibit expression of other factors. Cycloheximide activity was confirmed by the inhibition of GFP expression after treatment of J-Lat cells with TNF-α (Figure S6C). Under these conditions, aza-CdR still induced HIV-1 transcription (Figure S6D), indicating that aza-CdR acts directly upon the HIV-1 provirus. Synergistic reactivation of latent virus was not observed when aza-CdR was combined with the HDAC inhibitor valproic acid (VPA). Weak synergistic reactivation was observed when aza-CdR was combined with the HDAC inhibitor suberoylanilide hydroxamic acid (SAHA), with an effect about two-fold greater than the additive effect of the drugs (Figure S7).

We show in four different J-Lat cell lines that near-complete reactivation of latent HIV-1 required treatment with both an NF-κB activator and an inhibitor of DNA methylation (Figure 4D, lower panel). J-Lat A2 is another clone that harbors a latent HIV-derived vector encoding only the viral promoter and Tat. In contrast to the other cell lines analyzed here, latent virus in J-Lat A2 did not require aza-CdR for full reactivation. TNF-α alone reactivated the majority of latent virus in J-Lat A2 (Figures 4D, lower panel, and 4F) [33]. These data show that treatment with a methylation inhibitor is necessary for full reactivation of some, but not all, J-Lat cell lines.

### Cytosine methylation contributes to HIV-1 latency in a polyclonal cell population

**Figure 5:**
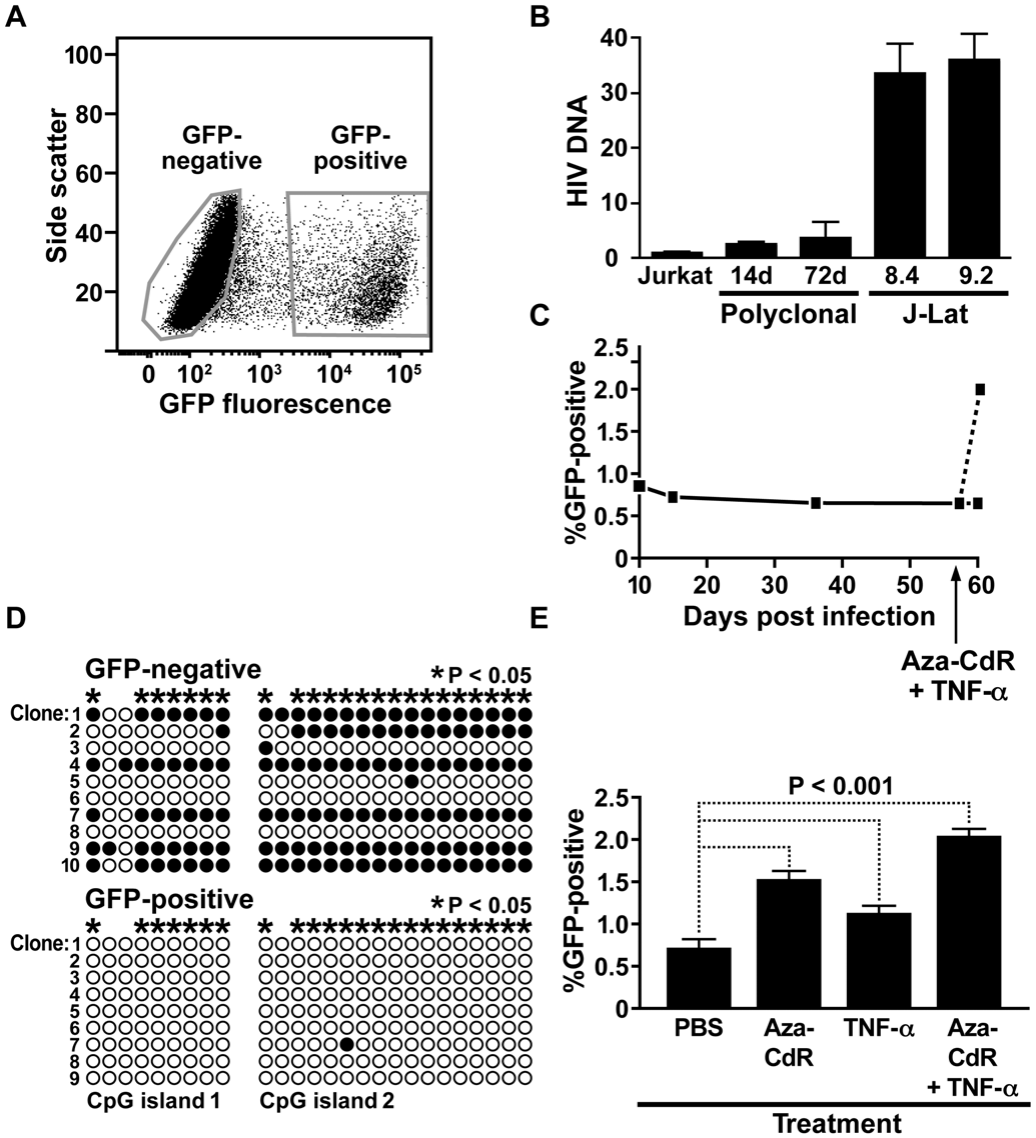
Cytosine methylation maintains HIV-1 latency in polyclonal Jurkat T cells. (A) Flow cytometry of Jurkat T cells infected with HIV-1 R7/E^−^/GFP clone. Gates indicate GFP-positive (productively infected) and GFP-negative (latently infected and uninfected) cells. (B) Quantitative PCR to measure HIV-1 DNA in infected Jurkat cells. For polyclonal cell populations, days after infection are indicated. For J-Lat clones, cell line is indicated. Levels of HIV-1 DNA were normalized to cellular DNA. Y-axis indicates fold over uninfected Jurkat negative control. Experiment was performed in triplicate and error bars indicate standard deviation. (C) HIV-1 expression over time in a polyclonal population of latently infected and uninfected Jurkat T cells. GFP fluorescence was measured by flow cytometry. The time point at which cells were treated with aza-CdR plus TNF-α is indicated on the x-axis. (D) Bisulfite-mediated methylcytosine mapping of HIV-1 CpG islands in polyclonal Jurkat T cells that are latently infected (GFP-negative, upper panel) or productively infected (GFP-positive, lower panel). Asterisks indicate cytosines with a statistically significant greater level of methylation in the GFP-negative population. (E) Latent HIV-1 reactivation in a polyclonal population of latently infected and uninfected Jurkat T cells treated with aza-CdR, TNF-α, or aza-CdR plus TNF-α. HIV-1 expression was measured by flow cytometry for GFP, and the percentage of cells that express GFP is displayed. Error bars indicate standard deviation of three experiments.

To confirm that cytosine methylation is regularly associated with HIV-1 latency, a polyclonal population of latently infected Jurkat T cells was generated by infection with virus produced from the R7/E^−^/GFP clone. All HIV-1 proteins are expressed from this full length HIV-1 molecular clone, except Nef, which is replaced with GFP, and Env, which is suppressed by a frameshift mutation. FACS was used to separate latently infected/uninfected GFP-negative cells from productively infected GFP-positive cells (Figure 5A). To compare the infection rate of this population to that of the J-Lat cells, quantitative PCR for HIV R7/E−/GFP sequence was performed on genomic DNA from the polyclonal population 14 and 72 days post infection. The quantity of HIV-1 DNA was normalized to cellular DNA using PCR primers that anneal upstream of the β-actin gene. The level of HIV DNA in these cells ranged from 9- to 14-fold less than that detected in J-Lat cells, indicating a lower rate of infection (Figure 5B). Bisulfite-mediated methylcytosine mapping of HIV-1 DNA from the productive population found hypomethylation, with no detectable methylation at most CpGs. In direct contrast, methylcytosine mapping of the latent population found hypermethylation, with the majority of CpGs methylated more than 68 percent of the time (Figure 5D and Figure S4F). In sodium bisulfite-treated DNA, cytosine was converted to thymine in greater than 99 percent of all CpN dinucleotides (N = A, T, or C), confirming efficient bisulfite conversion of non-methylated cytosines (Figure S5B).

Reactivation of latent HIV-1 was also examined in this population. After approximately two months, the proportion of cells with active HIV-1 remained stable at 0.65% (Figure 5C). Cells were then treated with TNF-α, aza-CdR, or TNF-α plus aza-CdR. TNF-α reactivated latent HIV-1, with a 1.5-fold greater proportion of cells with active virus (Figure 5E). Importantly, latent HIV-1 was also reactivated by aza-CdR alone, with a two-fold greater proportion of cells with active virus (Figure 5D). These observations indicate that, after infection of Jurkat cells *in vitro*, a subset of latently infected cells exists that can be reactivated solely by inhibition of DNA methylation.

### HIV-1 latency is associated with cytosine methylation in primary cells

The similarities of J-Lat cells to latently infected CD4^+^ T cells have established the utility of this experimental system for identifying and characterizing mechanisms of HIV-1 latency. However, because J-Lat cells divide autonomously and possess other aberrations associated with cellular transformation, cytosine methylation was analyzed in a recently developed primary cell model of latency [43]. In this system, naïve CD4^+^ T cells are purified from uninfected donors and activated under conditions that drive them to become memory cells with either a Th1, Th2, or non-polarized (NP) phenotype [44]. These differentiated cells are then infected with HIV-1 and viral expression is monitored. The phenotype of NP cells generated *ex vivo* (Figure S8) closely resembles that of central memory CD4^+^ T cells found *in vivo*, which persist for years in secondary lymphoid organs and can differentiate into effector memory CD4^+^ T cells [45]. A high rate of HIV-1 latency is observed in NP memory CD4^+^ T cells [43].

**Figure 6:**
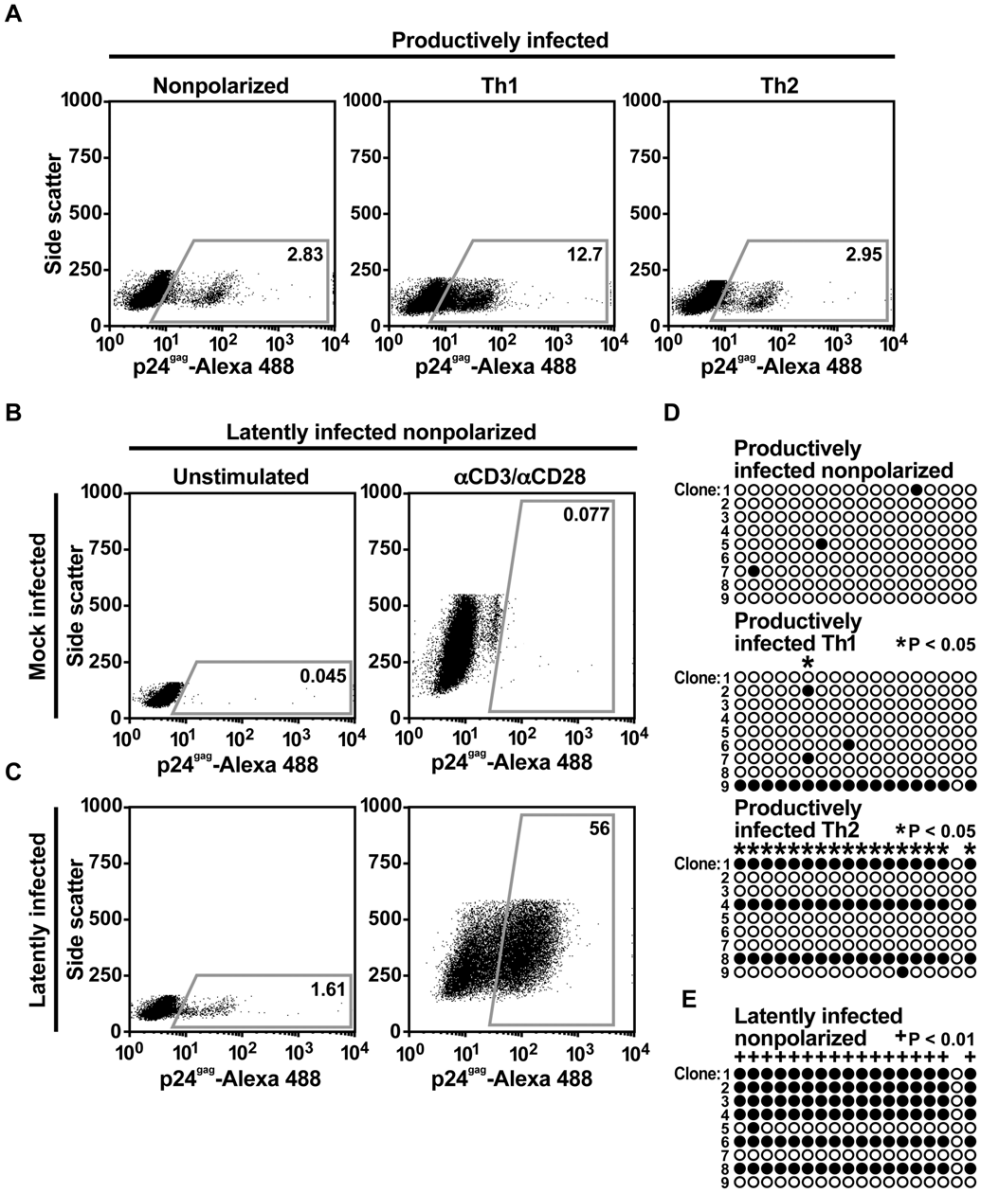
Cytosine methylation is associated with HIV-1 latency in primary CD4+ T cells. (A) Flow cytometric analysis of HIV-1 gene expression in CD4^+^ T cells productively infected under non-polarizing, Th1, or Th2 conditions. HIV-1 was detected by intracellular staining for p24^gag^. Gates indicate gag-positive cells, and the percentage of positive cells is indicated. (B,C) Flow cytometric analysis of HIV-1 gene expression in (B) mock infected or (C) latently infected CD4^+^ T cells under non-polarizing conditions, either at the basal state or after reactivation with antibodies against CD3 and CD28. HIV-1 was detected by intracellular staining for p24^gag^. Gates indicate gag-positive cells, and the percentage of gag-positive cells is indicated. (D) Bisulfite-mediated methylcytosine mapping of HIV-1 CpG island 2 in actively infected CD4^+^ T cells infected under non-polarizing, Th1, or Th2 conditions. (E) Bisulfite-mediated methylcytosine mapping of HIV-1 CpG island 2 in latently infected CD4^+^ T cells stimulated under non-polarizing conditions.

To determine if HIV-1 latency is associated with cytosine methylation in primary CD4^+^ T cells, bisulfite-mediated methylcytosine mapping was performed on CD4^+^ T cells activated under NP, Th1, and Th2 polarizing conditions and infected with HIV-1. Cells were infected with virus produced from the DHIV virus clone [46], in which CpG island 2 is conserved. Five days post-infection, p24^gag^ was detected in all three subsets (Figure 6A). At this early time point, the HIV-1 CpG island in the NP and Th1 populations was hypomethylated, with most CpGs methylated only 0 or 10 percent of the time, respectively (Figure 6D and Figure S4G). Significant methylation was detected in Th2 cells, with most CpGs methylated 33 percent of the time (Figure 6D and Figure S4G). Two weeks post-infection, NP cells had returned to a quiescent state and HIV-1 gene expression, as measured by intracellular p24^gag^ expression, was low (Figure 6C, left panel). However, stimulation with antibodies against CD3 and CD28 dramatically increased HIV-1 gene expression, indicating a large population of latently infected cells (Figure 6C, right panel). Importantly, CpG island methylation in latently infected NP cells was greater than in productively infected NP cells, with the majority of CpGs methylated 67 percent of the time (Figure 6E, S5D, and S4G). In sodium bisulfite-treated DNA, cytosine was converted to thymine in greater than 98 percent of all CpN dinucleotides (N = A, T, or C), confirming efficient bisulfite conversion of non-methylated cytosines (Figure S5C). These data confirm that T cell quiescence is associated with methylation of HIV-1 CpG islands and latency in memory CD4^+^ T cells.

## Discussion

Here, we describe a novel, phenotype-based screen to identify cellular proteins that control HIV-1 latency. This screen identified the transcriptional repressor MBD2 and led to the discovery that the latent HIV-1 provirus is hypermethylated in an *in vitro* model for HIV-1 latency and in primary lymphocytes latently infected with HIV-1. Based on these observations, we designed and tested a novel strategy for reactivation of latent HIV-1 using the synergistic activities of an inhibitor of cytosine methylation and activators of NF-κB signaling.

**Figure 7:**
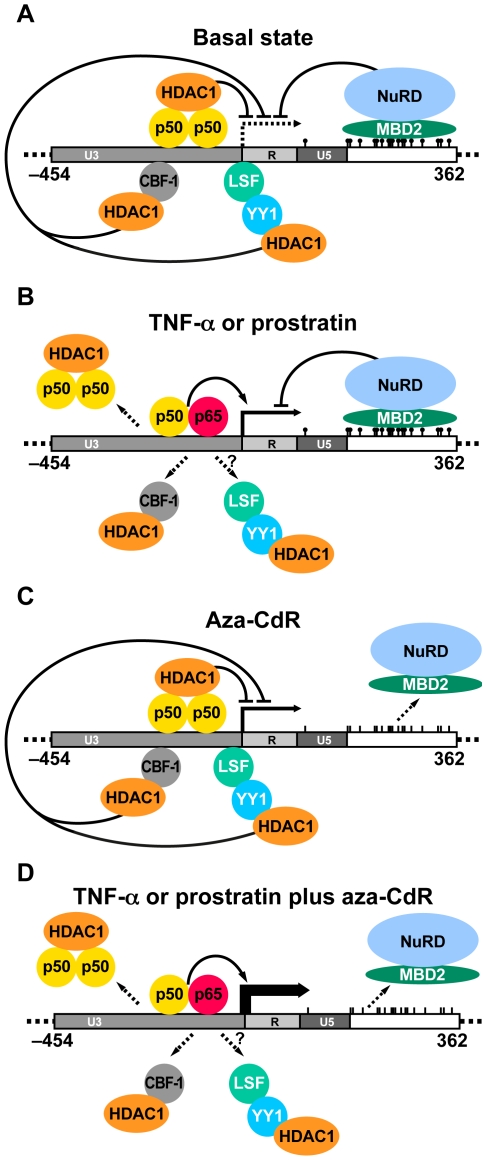
Molecular mechanisms of HIV-1 latency: Role of cytosine methylation. The first 821 nucleotides of the HIV-1 genome are shown, with genome position indicated relative to transcriptional start site. The virus promoter is comprised of the U3, R, and U5 regions. CpG island 2 is indicated by vertical lines, and filled circles on top of lines indicate methyl groups. Arrow indicates HIV-1 transcriptional start site. (A) Transcriptional repression during HIV-1 latency. Sequence-specific factors recruit HDAC1. CpG island 2 is methylated and bound by MBD2, which mediates transcriptional repression by recruitment of NuRD. (B) NF-κB activation triggers loss of p50 homodimers and CBF-1 from the HIV-1 promoter, resulting in decreased HDAC recruitment and partial reactivation of latent virus. Increased RelA (p65) recruitment has also been reported. Effects upon Yin-Yang 1 recruitment are unknown. (C) Treatment with aza-CdR decreases methylation of HIV-1 CpG islands, leading to a loss of MBD2 from the HIV-1 promoter. Latent virus is partially reactivated by removal of the methylation block. (D) Potent, synergistic reactivation of latent virus when NF-κB activation is combined with aza-CdR treatment. This occurs via loss of NF-κB-responsive transcriptional repressors and MBD2 from the HIV-1 promoter.

HIV-1 latency is likely to be a multifactorial process and a number of different mechanisms have been proposed to account for the establishment and the maintenance of the latent phenotype [13],[14],[20]. NF-κB signaling reactivates latent HIV [47]–[50], but data reported here and elsewhere [16],[51],[52] indicate that a significant proportion of latent HIV-1 remains silent when NF-κB is activated in the J-Lat clones or other cells. We show here that inhibiting provirus methylation leads to an almost complete reactivation of latent HIV-1 in the J-Lat cell lines when combined with activators of NF-κB. These data are consistent with the model that sequence-specific transcription factors and cytosine methylation cooperate to maintain HIV-1 latency. In the latent state, HDAC1 is recruited to the HIV-1 promoter by several sequence-specific factors including NF-κB p50 [18], CBF-1 [19], and Yin-Yang 1 [15]. Additionally, in microglial cells CTIP-2 has been shown to recruit HDAC1 to the HIV-1 promoter [53]. Our new observations demonstrate that MBD2 is also recruited to the latent HIV-1 promoter via the second CpG island (Figure 7A). We propose that MBD2 silences transcription by recruitment of the NuRD complex or other factors. This is supported by our finding that another component of NuRD, HDAC2, is also recruited to hypermethylated CpG island 2 during latency. NF-κB activation relieves one component of the transcriptional block, causing decreased CBF-1 [19] and NF-κB p50 homodimer recruitment to the HIV-1 promoter, as well as increased binding of the NF-κB RelA activator [18] (Figure 7B). Inhibition of cytosine methylation relieves another component of the transcriptional block, causing decreased MBD2 and HDAC2 recruitment to HIV-1 CpG island 2 (Figure 7C). The combination of NF-κB activation and methylation inhibitors eliminates both transcriptional blocks, causing a synergistic increase in HIV-1 transcription and reactivating virus in the majority of cells (Figure 7D).

In polyclonal Jurkat cells, the magnitude of HIV-1 reactivation appeared to be smaller than for the J-Lat clones. This was not, however, because TNF-α or aza-CdR were ineffective, but because of the small proportion of latently infected cells in this population compared to the J-Lat cells, each of which harbor a provirus. To ensure no more than one provirus per cell, they were infected at a low multiplicity that left approximately 90 percent of the cells uninfected. Quantitative PCR for HIV DNA demonstrated the small proportion of infected cells in this population compared to the J-Lat clones. Virus reactivation by TNF-α and aza-CdR is highly significant, but is somewhat obscured by the large background of GFP-negative uninfected cells.

The role of epigenetic mechanisms in suppression of HIV-1 transcription during latency has not been fully addressed to date. Sequence-specific transcription factors contribute to latency by recruiting HDACs and other repressors to the virus promoter. These findings present a paradox, however, because latent virus can be of wild-type nucleotide sequence [33], and yet transcription is suppressed. Here, we present evidence that HIV-1 latency is also maintained at the epigenetic level by the methylation of provirus DNA and recruitment of MBD2. This protein brings transcriptional repressors to methylated DNA, and the MBD2_1345–1947_ fragment isolated from the screen may reactivate latent HIV-1 by disrupting the interaction of MBD2 with an interaction partner. Importantly, after each round of DNA replication, cytosine methylation is faithfully reproduced in a process that is directed by previously methylated DNA [54]. Thus, identical DNA sequences can be either active or silenced depending on their methylation status. Our and previous findings suggest that both HDACs and cytosine methylation contribute to HIV-1 latency, in agreement with a growing body of evidence demonstrating cooperation between these two gene silencing mechanisms [55],[56].

The rarity of latently infected cells and the lack of a marker for latent HIV-1 infection necessitate the use of *in vitro* model systems for detailed studies of this process. Transformed cells such as the Jurkat line may show aberrant DNA methylation patterns at specific loci [57], possibly complicating analyses of cytosine methylation and HIV-1 latency. However, when Jurkat cells are infected with HIV-1 the proportion of cells that become latently infected vs. productively infected is small, suggesting that transformation does not result in the indiscriminate methylation and repression of HIV-1. Furthermore, high-resolution analysis of cytosine methylation in primary and transformed cells has found less aberrant methylation of CpG island promoters in transformed cells than had been previously hypothesized based on candidate gene studies [58]. Importantly, we confirmed the association between HIV-1 latency and cytosine methylation in a primary cell model of HIV-1 latency.

The findings reported here, based upon a near full-length HIV-1 with wild type LTR and Tat sequences, add to previous studies that have used mutated forms of the HIV-1 promoter to describe a role for methylation of HIV-1 DNA in latency [59]–[61]. One report, however, has described latent HIV-1-derived vectors, or “minigenomes,” that lack all virus genes except for Tat. J-Lat clone A2 harbors exactly such a minigenome. Importantly, these latent minigenomes are not methylated [62] and are almost fully reactivated by TNF-α treatment, unlike the full-length genome [33]. Apparently, screens to isolate latently infected clones produced very different results when mini- instead of full-length genomes were used. For the minigenomes, removal of virus genes and repositioning of Tat out of its normal genomic context are likely to have altered transcriptional control. This alteration may have influenced the type of latently infected cell recovered from the screen. The screens that produced the J-Lat cells also selected for mechanisms that silence HIV-1 within several days after infection. Other screens for cells that silence HIV-1 at later time points have identified additional silencing mechanisms [19].

Our results indicate that cytosine methylation can be an important component of HIV-1 latency. In the case of the full-length J-Lat clones, a high degree of cytosine methylation is detected during latency. In the case of minigenomes such as J-Lat A2 or that characterized by Pion et al, the persistent lack of methylation may permit efficient reactivation by TNF-α alone. Pion et al also describe a lack of cytosine methylation in latently infected PBMCs, but the large proportion of productively infected cells in the analyzed population complicates this assay.

Novel approaches are required to reactivate latent HIV-1 in infected persons. Therapies that interfere with cytosine methylation are attractive candidates to reactivate suppressed virus and purge the latent HIV-1 reservoir. In uninfected human subjects, aza-CdR causes decreased CpG island methylation and reactivation of a silenced gene [63],[64]. In HIV-infected individuals, a similar decrease in methylation should be attainable and could reactivate latent virus. Furthermore, mechanisms by which aza-CdR induces hypomethylation are well understood [65],[66] and this pharmaceutical is approved for use in humans. Aza-CdR acts directly upon the HIV-1 provirus, because it reactivates HIV-1 transcription in the presence of cycloheximide. HIV-1 was reactivated to a lesser extent in this experiment, and this could result from cellular toxicity or inhibition of an indirect component to reactivation. Any indirect component to HIV-1 reactivation would not, however, make aza-CdR any less effective a drug for reactivation of latent HIV-1 in humans. Aza-CdR synergizes with prostratin, a phorbol ester that triggers reactivation of latent HIV-1 in the absence of T cell activation and inhibits *de novo* virus infection [67]. Thus, the combination of aza-CdR and prostratin may reactivate latent HIV-1 while minimizing additional HIV-1 infection and side effects associated with T cell activation [29]. Therefore, the inclusion of cytosine methylation inhibitors in antiretroviral therapy could represent a significant step toward elimination of the latent HIV-1 reservoir and clearance of virus from infected patients.

## Materials and Methods

### Cell culture and drug treatment

Jurkat and J-Lat cells were cultured in RPMI (Invitrogen) with 5% FBS (Gemini Bio-Products) and 5% Fetalplex (Gemini Bio-Products). For analysis of virus reactivation by flow cytometry, aza-CdR (Sigma) and TNF-α (Biosource) treatments were for 24 h, after which medium was replaced. Reactivation was assayed after an additional 48 h. For ChIP and bisulfite-mediated methylcytosine mapping, cells were treated for 30 h with aza-CdR. For cycloheximide experiments, cells were treated for 24 hours, either with or without 40 ng/ml cycloheximide.

### Plasmid and cDNA library generation

20 µg of pEGFP-N1 (Clontech) was methylated at CpGs with M. *Sss* I (New England Biolabs) according to the manufacturer's protocol. DNA was purified and subjected to a second round of methylation. To generate pBMN-CSI-T, the multiple cloning site (MCS) and GFP gene from pBMN-I-GFP (Addgene plasmid 1736) were replaced with the MCS from pDNR-LIB (Clontech) and the tomato fluorescent protein. Also, the human cytomegalovirus (hCMV) immediate early promoter was inserted upstream of the MCS. For production of RelA-expressing retrovirus, RelA was cloned from pCMV4(hind), kindly provided by W. Greene, into a version of pBMN-CSI-T lacking the hCMV promoter. MBD2_1345–1947_ was cloned into pBMN-CSI-T as part of cDNA library generation. The cDNA library was generated using the Creator SMART cDNA Library Construction Kit (Clontech) with oligodT-purified (Quickprep mRNA Purification Kit, Amersham) RNA isolated (TRIzol, Invitrogen) from Jurkat T cells. Amplified cDNAs were cloned into pBMN-CSI-T and electroporated into *E. coli* strain DH5α. The library was amplified 240,000-fold by plating of bacteria on solid medium, and DNA was extracted from aliquots (Plasmid Maxi Kit, Qiagen).

### Plasmid transfection, cell infection, and screening

J-Lat cells were transfected by electroporation using Kit R and program O-28 (Amaxa Biosystems). HIV-1 reactivation was assayed by flow cytometry four days post-transfection. HIV-1 R7/E^−^/GFP pseudotyped with the vesicular stomatitus virus G (VSV-G) protein was produced by cotransfecting 293T cells with pEV1335 and a plasmid encoding VSV-G by the calcium phosphate method. Supernatant was harvested 48 h post-transfection and frozen at −80°C in aliquots. Aliquots were thawed, diluted 1∶160, and used to infect Jurkat T cells overnight at a multiplicity of 0.1 infectious units per cell with 2 ml supernatant per 1 million cells. Three days post-infection, GFP-negative and -positive cell populations were isolated by FACS. Retrovirus pseudotyped with VSV-G was produced as described previously [68] by cotransfection of Phoenix-ampho cells with pBMN-CSI-T or plasmids derived thereof and a plasmid encoding VSV-G. Supernatant was harvested 48 h post-transfection and J-Lat cells were infected overnight at a ratio of 250,000 cells to 2 ml supernatant with centrifugation at 2500 rpm for the first 1.5 h. 293T cells were transfected by the calcium phosphate method. Cells were cotransfected with a plasmid encoding the tomato fluorescent protein and either methylated or unmethylated pEGFP-N1, and the tomato-positive population was analyzed for GFP expression. For measurement of J-Lat activation, cells were infected with undiluted virus and analyzed by flow cytometry 2 days post-infection. For cDNA screening, cells were infected at a multiplicity of 0.15 infectious units per cell. GFP-positive cells were purified by FACS two days post-infection, cultured for two days, and genomic DNA was isolated (DNeasy Tissue Kit, Qiagen). The cDNA inserts were amplified from genomic DNA by PCR using oSK57 (5′-AAATGGGCGGTAGGCGTGTACGGTG-3′) and oSK58 (5′-GCGGCTTCGGCCAGTAACGTTAGGG-3′) as primers, cloned into pBMN-CSI-T, and identified by determination of nucleotide sequence (Molecular Cloning Laboratories).

### Flow cytometry and FACS

Cell fluorescence was measured with the FACSCalibur or LSRII (BD Biosciences). Cell sorting was performed with the FACS Vantage DiVa (BD Biosciences). To phenotype CD4^+^ T cells, they were stained with the following mAbs: phycoerythrin-conjugated (PE)–anti-CD4, TC–anti-CD45RA, or PE–anti-CXCR4 (Caltag). Flow cytometry and sorting data were analyzed with FlowJo software (Treestar) or Cellquest (BD Biosciences), in the case of primary cells. Analysis was restricted to the live population, as defined by the forward versus side scatter profile. Flowjo transforms fluorescence plots to a linear scale at the origin, permitting intelligible display of cells with low fluorescence.

To assess intracellular p24^gag^ expression, cells were fixed and permeabilized with Citofix/Cytoperm (BD Biosciences). Cells were washed with Perm/Wash buffer (BD Biosciences) and were stained with anti-p24 antibody (AG3.0). Cells were washed with Perm/Wash Buffer and incubated with Alexa Fluor 488 goat anti-mouse IgG (H+L) in 100 µl of Perm/Wash buffer. Cells were washed with Perm/Wash buffer and samples were analyzed by flow cytometry. HIV-1 p24^gag^ -positive gates were set by comparison with uninfected cells treated in parallel.

### Transfection of siRNAs, reverse transcription, and quantitative PCR

J-Lat cells were transfected with siRNAs corresponding to the MBD2 mRNA or non-targeting control siRNAs (siGENOME SMARTpool or siCONTROL pool, Dharmacon) by electroporation using Kit R and program O-28 (Amaxa Biosystems). Two days after transfection of siRNAs, RNA was isolated from cells with TRIzol Reagent (Invitrogen), treated with DNAse I (Promega), and first strand cDNA was synthesized with reverse transcriptase (Superscript II, Invitrogen) using a dT_16_ primer. Quantitative PCR was performed with the 7900HT Sequence Detection System (Applied Biosystems) and the 2× Hot Sybr real time PCR kit (Molecular Cloning Laboratories), with each PCR reaction receiving 1/20 of the reverse transcription. HIV R7/E^−^/GFP mRNA and DNA were assayed using oSK1 (5′-ATGGTGAGCAAGGGCGAGGAG-3′) and oSK5 (5′-GTGGTGCAGATGAACTTCAG-3′), oligonucleotides, corresponding to the GFP gene, as primers. HIV R7/E−/GFP DNA was normalized to a DNA sequence upstream of the human β-actin gene using 5USBACT (5′-GCCAGCTGCAAGCCTTGG-3′) and 3USBACT [18] (5′-GCCACTGGGCCTCCATTC-3′) as primers. MBD2 mRNA was assayed using oSK61 (5′-CCCACAACGAATGAATGAACAGC-3′) and oSK62 (5′-TGAAGACCTTTGGGTAGTTCCA-3′) as primers. As an internal control, HIV and MBD2 mRNA levels were normalized to that of cyclophilin A. Cyclophilin A mRNA was assayed using oSK6 (5′-GTCTCCTTTGAGCTGTTTGC-3′) and oSK7 (5′-CCATAGATGGACTTGCCACC-3′) as primers. IκB-α mRNA was assayed using oSK135 (5′- CTCCGAGACTTTCGAGGAAATAC-3′) and oSK136 (5′- GCCATTGTAGTTGGTAGCCTTCA-3′) as primers. SDS 2.3 software (Applied Biosystems) was used to quantify each cDNA relative to cyclophilin A and to confirm the specificity of each PCR reaction by melting curve analysis.

### Bisulfite-mediated methylcytosine mapping

Sodium bisulfite treatment was performed according to the Pikaard protocol (http://www.biology.wustl.edu/pikaard/PDFs%20and%20protocol%20files%20/Bisulfite%20Sequencing.pdf) with minor modifications. Jurkat T cell DNA was digested overnight with *Pst* I and purified with the Qiaquick PCR Purification Kit (Qiagen). Bisulfite-treated DNA was amplified in nested PCR reactions with the following reaction conditions: denature (95°C, 5 minutes), cycle 35 times (95°C, 30 seconds then 60°C, 60 seconds), and extend (60°C, 7 minutes). For J-Lat cells, the first reaction used oSK100 (5′-CGCCTCGAGTTTATTGATTTTTGGATGGTGTTAT-3′) and oSK101 (5′-CGCTCTAGACCATTTACCCCTAAATATTCTACAC-3′) as primers and the second reaction used oSK71 (5′-CGCCTCGAGATATTTTGTGAGTTTGTATGGGATG-3′) and oSK94 (5′-CGCTCTAGACCCAATATTTATCTACAA-3′) as primers. For primary cells infected with NL4-3-derived virus, the first reaction used oSK123 (5′-CGCCTCGAGTTTATTGATTTTTGGATGGTGTTTT-3′) and oSK124 (5′-CGCTCTAGACCATTTACCCCTAAAAATTCTACAC-3′) as primers and the second reaction used oSK122 (5′-CGCCTCGAGATATTTTATGAGTTAGTATGGGATG-3′) and oSK94 as primers. All PCR reactions were performed in triplicate and then pooled to reduce chances of clonality in recovered fragments. Products were cleaved with *Xho* I and *Xba* I and cloned into pBluescript (Stratagene) cleaved with the same enzymes. Nucleotide sequence was determined of at least nine cloned inserts using the universal M13 reverse primer. The efficiency of sodium bisulfite conversion was calculated using the Quantification Tool for Methylation Analysis (QUMA) software [69]. The nucleotide sequence of untreated DNA was also determined to ensure that readings do not result from virus mutations.

### Statistical analyses

The effect of MBD2_1345–1947_ upon GFP expression (Figure 2A) was evaluated by a two-tailed, two sample Student's t-test with a null hypothesis of no effect. Reactivation of latent HIV-1 (Figure 5D) was evaluated with a one-tailed, two sample Student's t-test with a null hypothesis of no increase in GFP expression. In bisulfite-mediated methylcytosine mapping experiments, at least nine independent clones of sodium bisulfite-treated HIV-1 DNA were analyzed from each sample. For J-Lat cell lines in the latent state (Figure 3B) and CD4^+^ T cells (Figures 6D and E), a one-tailed, single sample Student's t-test was performed for each CpG with a null hypothesis of no methylation. For J-Lat 6.3 treated with either aza-CdR or a PBS control (Figure 4A), a one-tailed, two-sample Student's t-test was performed for each CpG with a null hypothesis of no decrease in methylation after aza-CdR treatment. For sorted populations of GFP-negative and -positive Jurkat T cells (Figure 5B), a one-tailed, two sample Student's t-test was performed for each CpG with the null hypothesis that the GFP-positive population did not have less methylation.

### Chromatin immunoprecipitation and quantitative PCR

ChIP was performed as described previously [70] with modifications. J-Lat 6.3 cells Cells were diluted to 5×10^5^ per ml, lysed, and sonicated (Model 500 Ultrasonic Dismembranator, Fisher Scientific). Lysates were incubated overnight with 5 µg of antibody against MBD2 (Upstate Cell Signaling Solutions cat. 07-198), HDAC2 (Santa Cruz Biotechnology cat. sc-7899) or Sp1 (Santa Cruz Biotechnology cat. sc-59). Immune complexes were recovered by incubation for 1 h with protein A agarose beads (Invitrogen). Immunoprecipitated DNA was quantified by quantitative PCR using the 7900HT Sequence Detection System (Applied Biosystems) and 2× Hot Sybr real time PCR kit (Molecular Cloning Laboratories). Negative control DNA was assayed using 5USBACT and 3USBACT as primers, CpG island 1 was assayed using oSK92 (5′-TCAGTTCAGATAATTTCAGTTGTCC-3′) and oSK93 (5′-CCCAGTACAGGCAAAAAGCA-3′) as primers, and CpG island 2 was assayed using oSK89 (5′-AAGCGAAAGGGAAACCAGAG-3′) and oSK90 (5′-TCTCCCCCGCTTAATACTGA-3′) as primers. SDS 2.3 software (Applied Biosystems) was used to analyze precipitated DNA relative to input and to confirm the specificity of each PCR reaction by melting curve analysis.

### Differentiation, infection, and activation of CD4^+^ T cells ex vivo

PBMCs were obtained from leukopaks from unidentified, healthy donors. Naïve CD4^+^ T cells were isolated by MACS microbead negative sorting using the naïve T cell isolation kit (Miltenyi Biotec). The purity of the population was always higher than 95%. Naïve T cells were primed with beads coated with anti-CD3 and anti-CD28 (Dynal/Invitrogen) as previously described [44].

Seven days after stimulation, cells were infected by spinoculation. Seven days after infection, cells were reactivated with beads coated with anti-CD3 and anti-CD28 for 72 h in the presence of IL-2 at a ratio of 1 bead per cell. The integrase inhibitor 118-D-24 did not have any effect on viral reactivation.

## Supporting Information

Figure S1 Recovery and analysis of MBD2 fragment. (A) Products of PCR amplification using template DNA from J-Lat 6.3 cells. Cells were infected with indicated viruses. For GFP^+^, DNA was isolated from GFP-positive cells 4 days post-infection with the cDNA library. The PCR product corresponding to MBD2_1345–1947_ is indicated. (B) Electrophoresis of unmethylated or methylated pEGFP-N1 after incubation with restriction enzyme *Msp* I (methylation-insensitive) or *Hpa* II (methylation-sensitive).
(1.53 MB TIF)

Figure S2 Genetic structure of cloned MBD2 fragment. Genetic structure of MBD2_1345–1947_ clone recovered from screen and full-length MBD2 mRNA. MBD2_1345–1947_ was cloned into pBMN-CSI-T as part of cDNA library generation. Dashed lines indicate portion of MBD2 mRNA cloned into pBMN-CSI-T. Open inverted triangles indicate predicted translation initiation codons. Closed inverted triangles indicate translation stop codons. GR, glycine-arginine repeat region; MBD, methyl-binding domain; TR, transcriptional repression domain.
(0.22 MB TIF)

Figure S3 CpG islands flanking the HIV-1 transcriptional start site. Nucleotide sequence of first 1000 bases of HIV-1 strain HXB2 provirus. Locations of CpG islands 1 and 2 are indicated and highlighted in yellow. Methylation status of CpGs in bold was determined with bisulfite-mediated methylcytosine mapping. The U5, R, and U3 regions of the HIV-1 promoter are indicated. Translation initiation site of Gag polyprotein is indicated. Only CpG island 2 is conserved in HIV-1 strain NL4-3.
(1.33 MB TIF)

Figure S4 HIV-1 CpG islands are methylated during latency Level of HIV-1 cytosine methylation in J-Lat cell line (A) 6.3, (B) 8.4, (C) 9.2, or (D) 15.4. Data points represent the frequency of methylation detected for each CpG within the analyzed region. (E) Frequency of HIV-1 cytosine methylation in J-Lat 6.3 treated with aza-CdR or PBS as a control. (F) Frequency of HIV-1 cytosine methylation in purified GFP-negative and -positive populations after infection of cells with HIV-1 R7/E−/GFP clone. (G) Frequency of HIV-1 cytosine methylation in latently infected nonpolarized CD4^+^ T cells or productively infected Th1, Th2, or nonpolarized CD4^+^ T cells. Data points correspond to the frequency of methylation detected for each CpG within the analyzed region.
(1.13 MB TIF)

Figure S5 Sodium bisulfite conversion is highly efficient Mean percentage of cytosine-to-thymine conversion in non-CpG dinucleotides by sodium bisulfite treatment of (A) J-Lat cells, (B) Jurkat cells infected with HIV-1 R7/E−/GFP clone, or (C) CD4^+^ T cells. Error bars indicate standard deviation. (D) Results of bisulfite-mediated methylcytosine mapping of HIV-1 CpG island 2 for additional clones of latently infected CD4^+^ T cells stimulated under non-polarizing conditions.
(0.62 MB TIF)

Figure S6 Synergistic activation of transcription is specific for the HIV-1 promoter. Steady-state mRNA levels were measured in (A) J-Lat 8.4 or (B) J-Lat 9.2. Quantity of HIV-1 (left panel) and IKB-α (right panel) mRNA was determined by reverse transcription and quantitative PCR after indicated treatments. Values are normalized to the PBS control. Error bars indicate standard deviation of quantitative PCR results. (C) Flow cytometric analysis of GFP expression in J-Lat cells after treatment with TNF-α, either in the presence or absence of cycloheximide. Histograms indicate GFP fluorescence. Gates indicate GFP-positive cells. (D) Levels of or HIV-1 (right panel) mRNA were determined by reverse transcription and quantitative PCR and normalized to cyclophilin mRNA. Cells were treated with aza-CdR, either in the presence or absence of cycloheximide. Error bars indicate standard deviation of qPCR results.
(0.79 MB TIF)

Figure S7 Aza-CdR and HDAC inhibitors do not synergistically reactivate latent HIV-1 Latent HIV-1 reactivation in the indicated J-Lat cell lines treated with aza-CdR, VPA, aza-CdR plus VPA, SAHA, or aza-CdR plus SAHA. GFP fluorescence was measured by flow cytometry and normalized to control cells treated with DMSO. Experiments were performed in triplicate and error bars indicate standard deviation.
(0.32 MB TIF)

Figure S8 Phenotypic analysis of CD4^+^ T cells. Flow cytometric analysis of two donors, (A) and (B), is shown. Expression of CD4 (helper T cell), CD45RA (naïve T cell), CD69 (early activation), and CD25 (late activation) markers was determined after isolation and stimulation of naïve T cells. Grey histogram represents cells incubated with a non-fluorescent isotype control antibody. Data are representative of those from five different donors.
(0.72 MB TIF)

Table S1 Reactivation of latent HIV-1.
(0.04 MB DOC)

## References

[1] Gulick RM, Mellors JW, Havlir D, Eron JJ, Gonzalez C (1997). Treatment with indinavir, zidovudine, and lamivudine in adults with human immunodeficiency virus infection and prior antiretroviral therapy.. N Engl J Med.

[2] Hammer SM, Squires KE, Hughes MD, Grimes JM, Demeter LM (1997). A controlled trial of two nucleoside analogues plus indinavir in persons with human immunodeficiency virus infection and CD4 cell counts of 200 per cubic millimeter or less. AIDS Clinical Trials Group 320 Study Team.. N Engl J Med.

[3] Perelson AS, Essunger P, Cao Y, Vesanen M, Hurley A (1997). Decay characteristics of HIV-1-infected compartments during combination therapy.. Nature.

[4] Palella FJ, Delaney KM, Moorman AC, Loveless MO, Fuhrer J (1998). Declining morbidity and mortality among patients with advanced human immunodeficiency virus infection. HIV Outpatient Study Investigators.. N Engl J Med.

[5] Chun TW, Carruth L, Finzi D, Shen X, DiGiuseppe JA (1997). Quantification of latent tissue reservoirs and total body viral load in HIV-1 infection.. Nature.

[6] Chun TW, Stuyver L, Mizell SB, Ehler LA, Mican JA (1997). Presence of an inducible HIV-1 latent reservoir during highly active antiretroviral therapy.. Proc Natl Acad Sci U S A.

[7] Finzi D, Hermankova M, Pierson T, Carruth LM, Buck C (1997). Identification of a reservoir for HIV-1 in patients on highly active antiretroviral therapy.. Science.

[8] Wong JK, Hezareh M, Gunthard HF, Havlir DV, Ignacio CC (1997). Recovery of replication-competent HIV despite prolonged suppression of plasma viremia.. Science.

[9] Chun TW, Davey RT, Engel D, Lane HC, Fauci AS (1999). Re-emergence of HIV after stopping therapy.. Nature.

[10] Chun TW, Davey RT, Ostrowski M, Shawn Justement J, Engel D (2000). Relationship between pre-existing viral reservoirs and the re-emergence of plasma viremia after discontinuation of highly active anti-retroviral therapy.. Nat Med.

[11] Zhang L, Chung C, Hu BS, He T, Guo Y (2000). Genetic characterization of rebounding HIV-1 after cessation of highly active antiretroviral therapy.. J Clin Invest.

[12] Forsdyke DR (1991). Programmed activation of T-lymphocytes. A theoretical basis for short term treatment of AIDS with azidothymidine.. Medical Hypotheses.

[13] Geeraert L, Kraus G, Pomerantz RJ (2008). Hide-and-seek: the challenge of viral persistence in HIV-1 infection.. Annu Rev Med.

[14] Pierson T, McArthur J, Siliciano RF (2000). Reservoirs for HIV-1: mechanisms for viral persistence in the presence of antiviral immune responses and antiretroviral therapy.. Annu Rev Immunol.

[15] Coull JJ, Romerio F, Sun JM, Volker JL, Galvin KM (2000). The human factors YY1 and LSF repress the human immunodeficiency virus type 1 long terminal repeat via recruitment of histone deacetylase 1.. J Virol.

[16] du Chene I, Basyuk E, Lin YL, Triboulet R, Knezevich A (2007). Suv39H1 and HP1gamma are responsible for chromatin-mediated HIV-1 transcriptional silencing and post-integration latency.. Embo J.

[17] Marban C, Redel L, Suzanne S, Van Lint C, Lecestre D (2005). COUP-TF interacting protein 2 represses the initial phase of HIV-1 gene transcription in human microglial cells.. Nucleic Acids Res.

[18] Williams SA, Chen LF, Kwon H, Ruiz-Jarabo CM, Verdin E (2006). NF-kappaB p50 promotes HIV latency through HDAC recruitment and repression of transcriptional initiation.. Embo J.

[19] Tyagi M, Karn J (2007). CBF-1 promotes transcriptional silencing during the establishment of HIV-1 latency.. Embo J.

[20] Lassen K, Han Y, Zhou Y, Siliciano J, Siliciano RF (2004). The multifactorial nature of HIV-1 latency.. Trends Mol Med.

[21] Han Y, Lassen K, Monie D, Sedaghat AR, Shimoji S (2004). Resting CD4+ T cells from human immunodeficiency virus type 1 (HIV-1)-infected individuals carry integrated HIV-1 genomes within actively transcribed host genes.. J Virol.

[22] Lewinski MK, Bisgrove D, Shinn P, Chen H, Hoffmann C (2005). Genome-wide analysis of chromosomal features repressing human immunodeficiency virus transcription.. J Virol.

[23] Lenasi T, Contreras X, Peterlin BM (2008). Transcriptional interference antagonizes proviral gene expression to promote HIV latency.. Cell Host Microbe.

[24] Han Y, Lin YB, An W, Xu J, Yang HC (2008). Orientation-dependent regulation of integrated HIV-1 expression by host gene transcriptional readthrough.. Cell Host Microbe.

[25] Jordan A, Defechereux P, Verdin E (2001). The site of HIV-1 integration in the human genome determines basal transcriptional activity and response to Tat transactivation.. Embo J.

[26] Lassen KG, Ramyar KX, Bailey JR, Zhou Y, Siliciano RF (2006). Nuclear retention of multiply spliced HIV-1 RNA in resting CD4+ T cells.. PLoS Pathog.

[27] Huang J, Wang F, Argyris E, Chen K, Liang Z (2007). Cellular microRNAs contribute to HIV-1 latency in resting primary CD4+ T lymphocytes.. Nat Med.

[28] Stellbrink HJ, van Lunzen J, Westby M, O'Sullivan E, Schneider C (2002). Effects of interleukin-2 plus highly active antiretroviral therapy on HIV-1 replication and proviral DNA (COSMIC trial).. Aids.

[29] Prins JM, Jurriaans S, van Praag RM, Blaak H, van Rij R (1999). Immuno-activation with anti-CD3 and recombinant human IL-2 in HIV-1-infected patients on potent antiretroviral therapy.. Aids.

[30] van Praag RM, Prins JM, Roos MT, Schellekens PT, Ten Berge IJ (2001). OKT3 and IL-2 treatment for purging of the latent HIV-1 reservoir in vivo results in selective long-lasting CD4+ T cell depletion.. J Clin Immunol.

[31] Siliciano JD, Lai J, Callender M, Pitt E, Zhang H (2007). Stability of the Latent Reservoir for HIV-1 in Patients Receiving Valproic Acid.. The Journal of Infectious Diseases.

[32] Steel A, Clark S, Teo I, Shaunak S, Nelson M (2006). No change to HIV-1 latency with valproate therapy.. Aids.

[33] Jordan A, Bisgrove D, Verdin E (2003). HIV reproducibly establishes a latent infection after acute infection of T cells in vitro.. Embo J.

[34] Fenaux P (2005). Inhibitors of DNA methylation: beyond myelodysplastic syndromes.. Nat Clin Pract Oncol.

[35] Wade PA (2001). Methyl CpG-binding proteins and transcriptional repression.. Bioessays.

[36] Bird AP, Wolffe AP (1999). Methylation-Induced Repression– Belts, Braces, and Chromatin.. Cell.

[37] Zhang Y, Ng HH, Erdjument-Bromage H, Tempst P, Bird A (1999). Analysis of the NuRD subunits reveals a histone deacetylase core complex and a connection with DNA methylation.. Genes Dev.

[38] Antequera F (2003). Structure, function and evolution of CpG island promoters.. Cell Mol Life Sci.

[39] Li LC, Dahiya R (2002). MethPrimer: designing primers for methylation PCRs.. Bioinformatics.

[40] Verdin E, Paras P, Van Lint C (1993). Chromatin disruption in the promoter of human immunodeficiency virus type 1 during transcriptional activation.. Embo J.

[41] el Kharroubi A, Verdin E (1994). Protein-DNA interactions within DNase I-hypersensitive sites located downstream of the HIV-1 promoter.. Journal of Biological Chemistry.

[42] Lopez-Serra L, Esteller M (2008). Proteins that bind methylated DNA and human cancer: reading the wrong words.. Br J Cancer.

[43] Bosque A, Planelles V (2008). Induction of HIV-1 latency and reactivation in primary memory CD4+ T cells..

[44] Messi M, Giacchetto I, Nagata K, Lanzavecchia A, Natoli G (2003). Memory and flexibility of cytokine gene expression as separable properties of human T(H)1 and T(H)2 lymphocytes.. Nat Immunol.

[45] Sallusto F, Lenig D, Forster R, Lipp M, Lanzavecchia A (1999). Two subsets of memory T lymphocytes with distinct homing potentials and effector functions.. Nature.

[46] Andersen JL, DeHart JL, Zimmerman ES, Ardon O, Kim B (2006). HIV-1 Vpr-induced apoptosis is cell cycle dependent and requires Bax but not ANT.. PLoS Pathog.

[47] Brooks DG, Arlen PA, Gao L, Kitchen CM, Zack JA (2003). Identification of T cell-signaling pathways that stimulate latent HIV in primary cells.. Proc Natl Acad Sci U S A.

[48] Folks TM, Clouse KA, Justement J, Rabson A, Duh E (1989). Tumor necrosis factor alpha induces expression of human immunodeficiency virus in a chronically infected T-cell clone.. Proc Natl Acad Sci U S A.

[49] West MJ, Lowe AD, Karn J (2001). Activation of Human Immunodeficiency Virus Transcription in T Cells Revisited: NF-{kappa}B p65 Stimulates Transcriptional Elongation.. The Journal of Virology.

[50] Williams SA, Chen LF, Kwon H, Fenard D, Bisgrove D (2004). Prostratin antagonizes HIV latency by activating NF-kappaB.. J Biol Chem.

[51] Kim YK, Bourgeois CF, Pearson R, Tyagi M, West MJ (2006). Recruitment of TFIIH to the HIV LTR is a rate-limiting step in the emergence of HIV from latency.. Embo J.

[52] Williams SA, Kwon H, Chen LF, Greene WC (2007). Sustained induction of NF-kappa B is required for efficient expression of latent human immunodeficiency virus type 1.. J Virol.

[53] Marban C, Suzanne S, Dequiedt F, de Walque S, Redel L (2007). Recruitment of chromatin-modifying enzymes by CTIP2 promotes HIV-1 transcriptional silencing.. Embo J.

[54] Ooi SK, Bestor TH (2008). Cytosine methylation: remaining faithful.. Curr Biol.

[55] Weber M, Hellmann I, Stadler MB, Ramos L, Paabo S (2007). Distribution, silencing potential and evolutionary impact of promoter DNA methylation in the human genome.. Nat Genet.

[56] Vaissiere T, Sawan C, Herceg Z (2008). Epigenetic interplay between histone modifications and DNA methylation in gene silencing.. Mutation Research/Reviews in Mutation Research In Press, Corrected Proof.

[57] Rountree MR, Bachman KE, Herman JG, Baylin SB (2001). DNA methylation, chromatin inheritance, and cancer.. Oncogene.

[58] Weber M, Davies JJ, Wittig D, Oakeley EJ, Haase M (2005). Chromosome-wide and promoter-specific analyses identify sites of differential DNA methylation in normal and transformed human cells.. Nat Genet.

[59] Ishida T, Hamano A, Koiwa T, Watanabe T (2006). 5′ long terminal repeat (LTR)-selective methylation of latently infected HIV-1 provirus that is demethylated by reactivation signals.. Retrovirology.

[60] Jeeninga RE, Westerhout EM, van Gerven ML, Berkhout B (2008). HIV-1 latency in actively dividing human T cell lines.. Retrovirology.

[61] Obrien MC, Ueno T, Jahan N, Zajackaye M, Mitsuya H (1995). HIV-1 Expression Induced by Anticancer Agents in Latently HIV-1-Infected ACH2 Cells.. Biochemical and Biophysical Research Communications.

[62] Pion M, Jordan A, Biancotto A, Dequiedt F, Gondois-Rey F (2003). Transcriptional suppression of in vitro-integrated human immunodeficiency virus type 1 does not correlate with proviral DNA methylation.. J Virol.

[63] Saunthararajah Y, Hillery CA, Lavelle D, Molokie R, Dorn L (2003). Effects of 5-aza-2′-deoxycytidine on fetal hemoglobin levels, red cell adhesion, and hematopoietic differentiation in patients with sickle cell disease.. Blood.

[64] Mund C, Hackanson B, Stresemann C, Lubbert M, Lyko F (2005). Characterization of DNA Demethylation Effects Induced by 5-Aza-2′-Deoxycytidine in Patients with Myelodysplastic Syndrome.. Cancer Research.

[65] Christman JK (2002). 5-Azacytidine and 5-aza-2′-deoxycytidine as inhibitors of DNA methylation: mechanistic studies and their implications for cancer therapy.. Oncogene.

[66] Ghoshal K, Datta J, Majumder S, Bai S, Kutay H (2005). 5-Aza-deoxycytidine induces selective degradation of DNA methyltransferase 1 by a proteasomal pathway that requires the KEN box, bromo-adjacent homology domain, and nuclear localization signal.. Mol Cell Biol.

[67] Korin YD, Brooks DG, Brown S, Korotzer A, Zack JA (2002). Effects of prostratin on T-cell activation and human immunodeficiency virus latency.. J Virol.

[68] Pear WS, Nolan GP, Scott ML, Baltimore D (1993). Production of high-titer helper-free retroviruses by transient transfection.. Proc Natl Acad Sci U S A.

[69] Kumaki Y, Oda M, Okano M (2008). QUMA: quantification tool for methylation analysis.. Nucleic Acids Research.

[70] Yu J, Angelin-Duclos C, Greenwood J, Liao J, Calame K (2000). Transcriptional repression by blimp-1 (PRDI-BF1) involves recruitment of histone deacetylase.. Mol Cell Biol.

