# Supplementary figures and images for: Epigenetic Regulation of HIV-1 Latency by Cytosine Methylation
Source: PLoS Pathog. 2009 Jun 26;5(6):e1000495. doi: 10.1371/journal.ppat.1000495 (PMC2695767; doi:10.1371/journal.ppat.1000495)

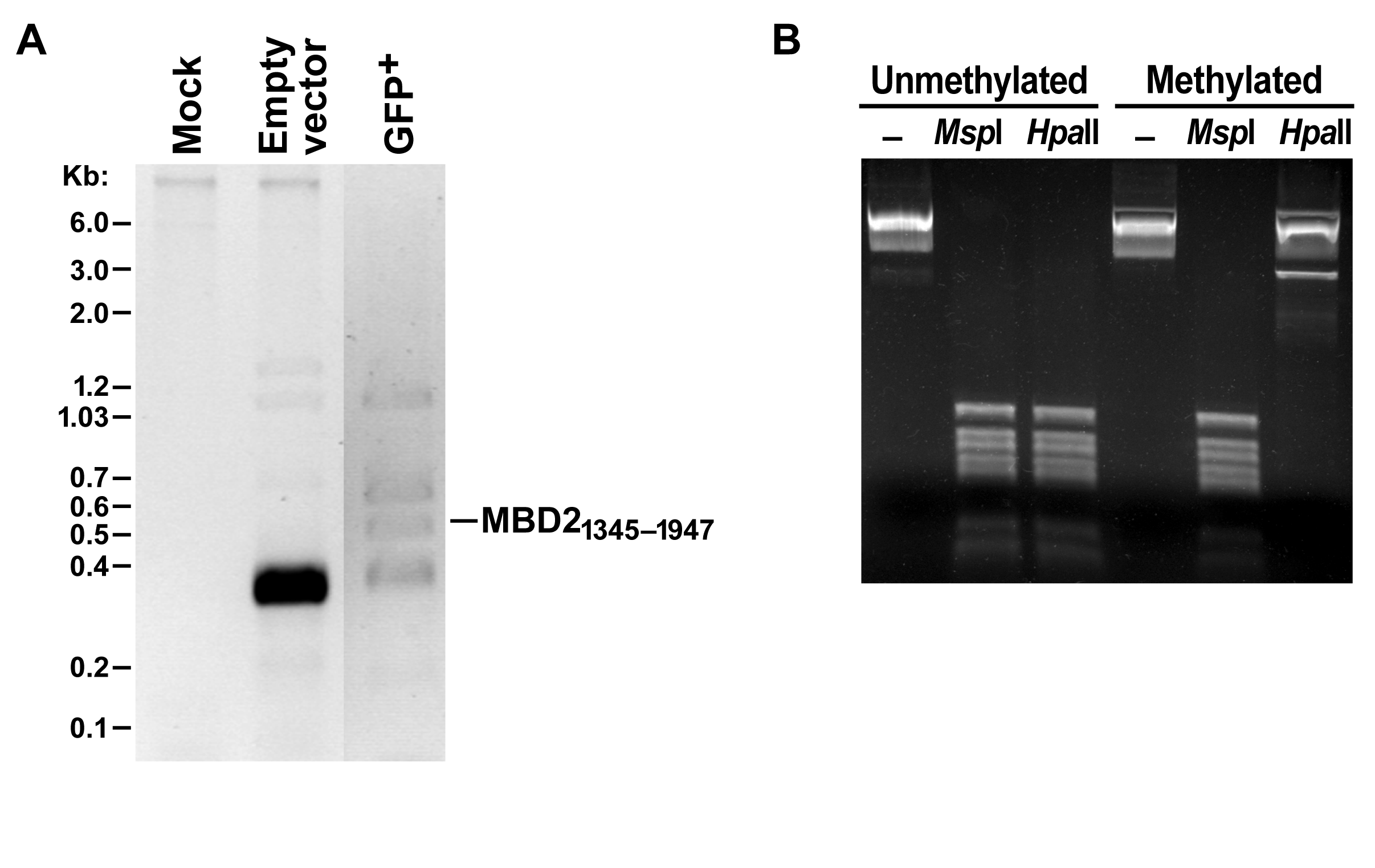

Supplement: Figure S1 — Recovery and analysis of MBD2 fragment. (A) Products of PCR amplification using template DNA from J-Lat 6.3 cells. Cells were infected with indicated viruses. For GFP+, DNA was isolated from GFP-positive cells 4 days post-infection with the cDNA library. The PCR product corresponding to MBD21345–1947 is indicated. (B) Electrophoresis of unmethylated or methylated pEGFP-N1 after incubation with restriction enzyme Msp I (methylation-insensitive) or Hpa II (methylation-sensitive). (1.53 MB TIF) [file ppat.1000495.s001.tif]

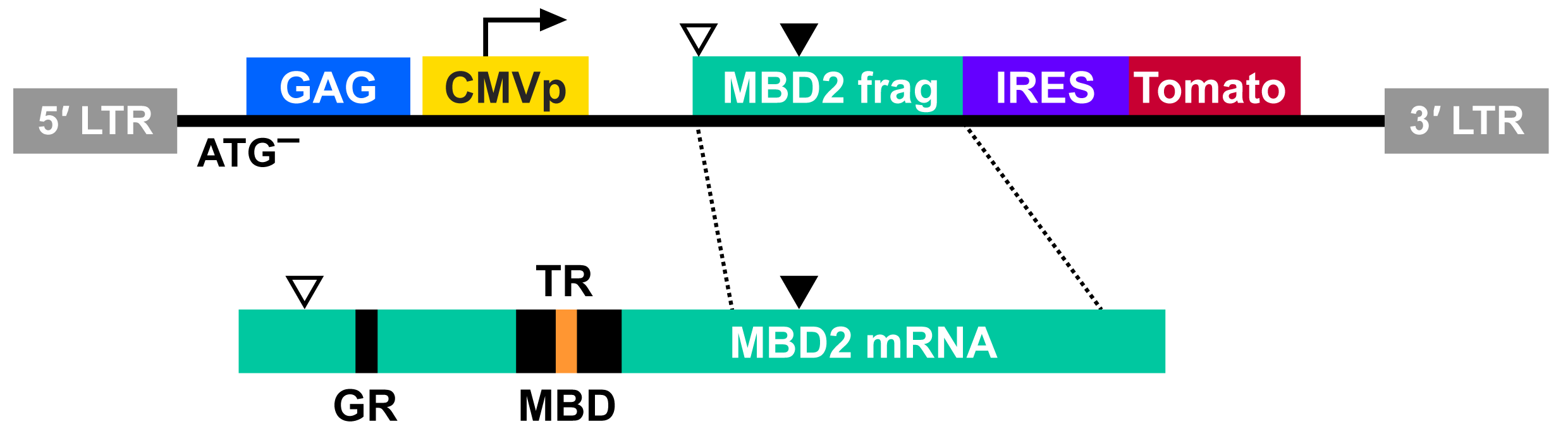

Supplement: Figure S2 — Genetic structure of cloned MBD2 fragment. Genetic structure of MBD21345–1947 clone recovered from screen and full-length MBD2 mRNA. MBD21345–1947 was cloned into pBMN-CSI-T as part of cDNA library generation. Dashed lines indicate portion of MBD2 mRNA cloned into pBMN-CSI-T. Open inverted triangles indicate predicted translation initiation codons. Closed inverted triangles indicate translation stop codons. GR, glycine-arginine repeat region; MBD, methyl-binding domain; TR, transcriptional repression domain. (0.22 MB TIF) [file ppat.1000495.s002.tif]

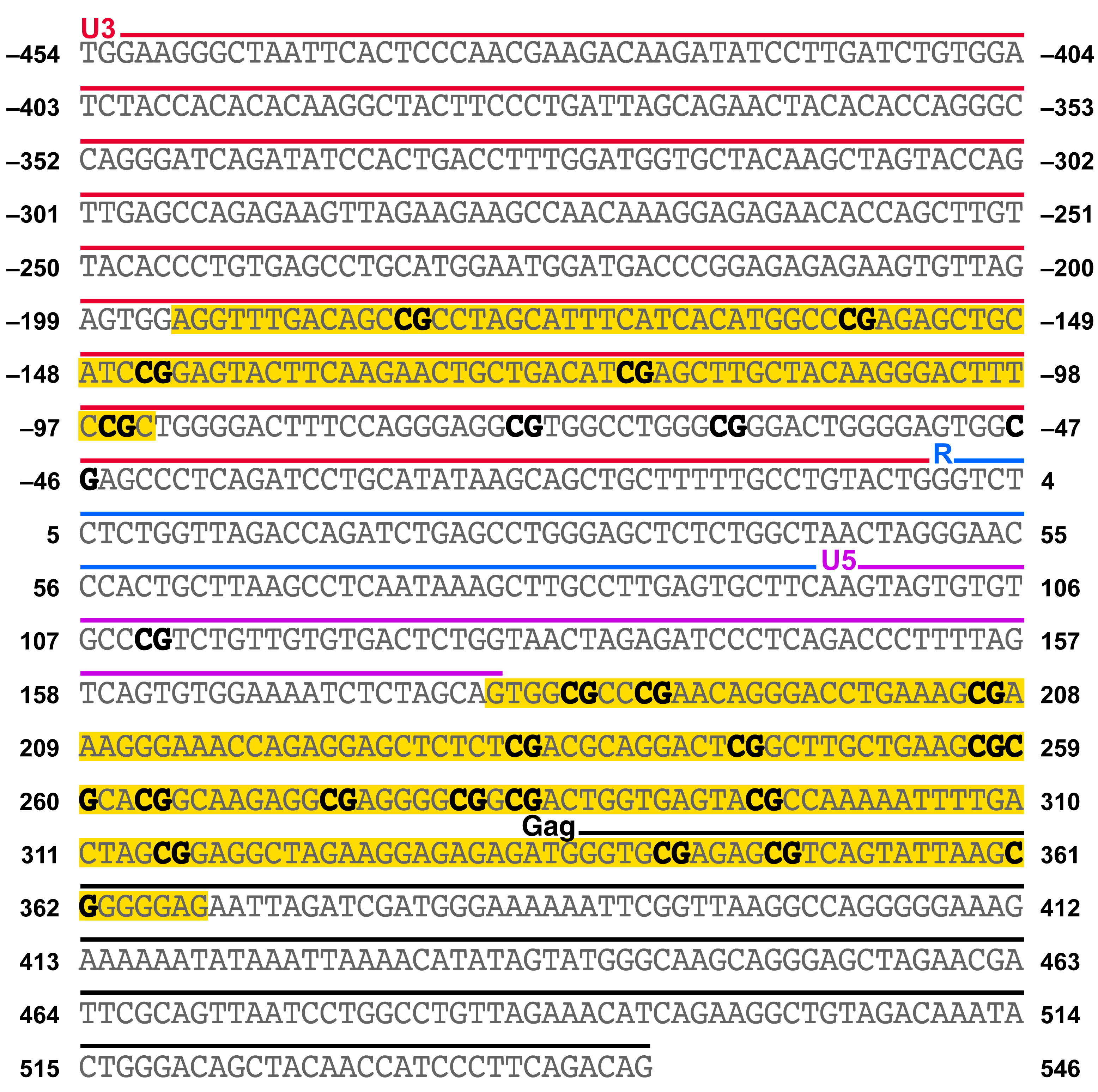

Supplement: Figure S3 — CpG islands flanking the HIV-1 transcriptional start site. Nucleotide sequence of first 1000 bases of HIV-1 strain HXB2 provirus. Locations of CpG islands 1 and 2 are indicated and highlighted in yellow. Methylation status of CpGs in bold was determined with bisulfite-mediated methylcytosine mapping. The U5, R, and U3 regions of the HIV-1 promoter are indicated. Translation initiation site of Gag polyprotein is indicated. Only CpG island 2 is conserved in HIV-1 strain NL4-3. (1.33 MB TIF) [file ppat.1000495.s003.tif]

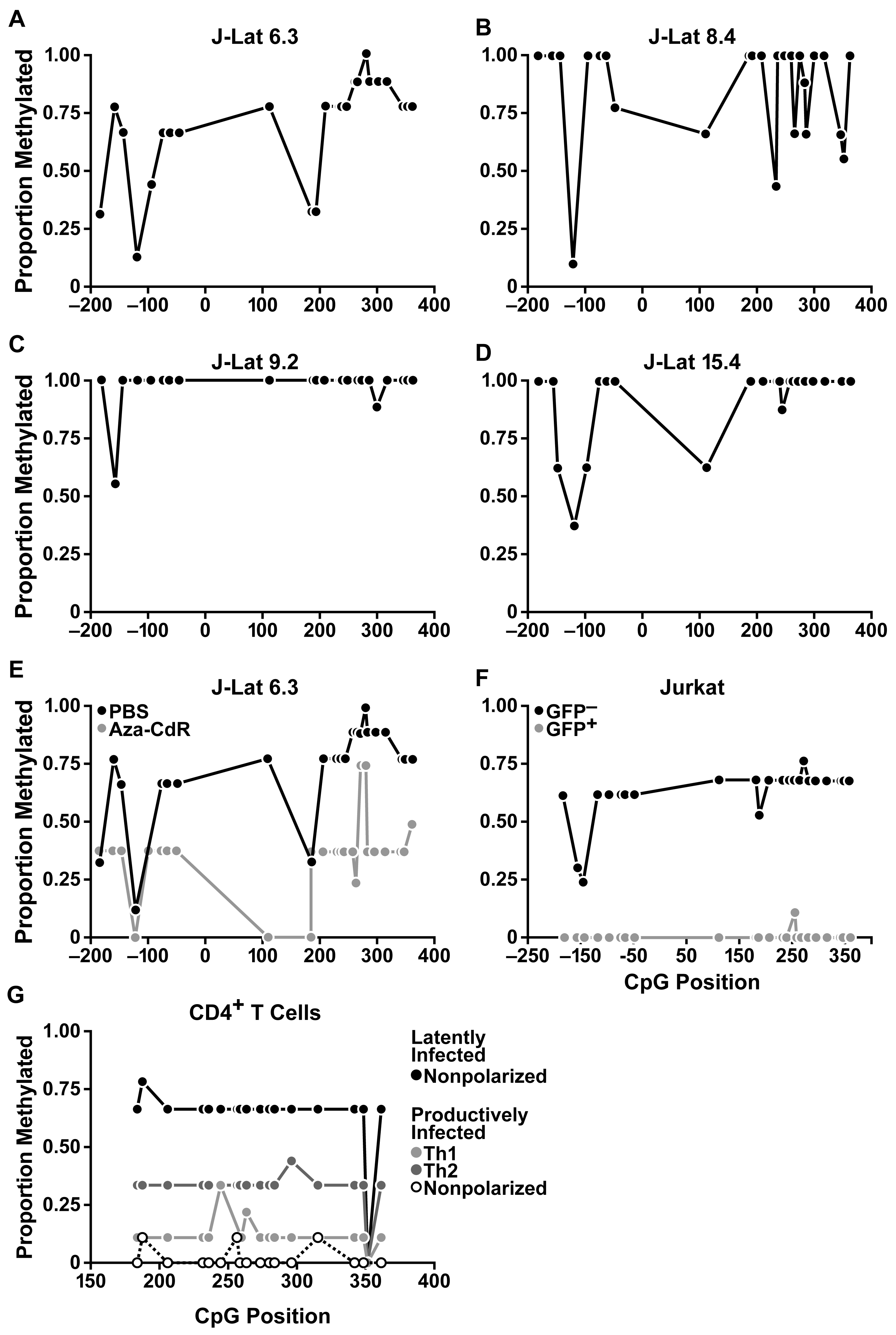

Supplement: Figure S4 — HIV-1 CpG islands are methylated during latency Level of HIV-1 cytosine methylation in J-Lat cell line (A) 6.3, (B) 8.4, (C) 9.2, or (D) 15.4. Data points represent the frequency of methylation detected for each CpG within the analyzed region. (E) Frequency of HIV-1 cytosine methylation in J-Lat 6.3 treated with aza-CdR or PBS as a control. (F) Frequency of HIV-1 cytosine methylation in purified GFP-negative and -positive populations after infection of cells with HIV-1 R7/E−/GFP clone. (G) Frequency of HIV-1 cytosine methylation in latently infected nonpolarized CD4+ T cells or productively infected Th1, Th2, or nonpolarized CD4+ T cells. Data points correspond to the frequency of methylation detected for each CpG within the analyzed region. (1.13 MB TIF) [file ppat.1000495.s004.tif]

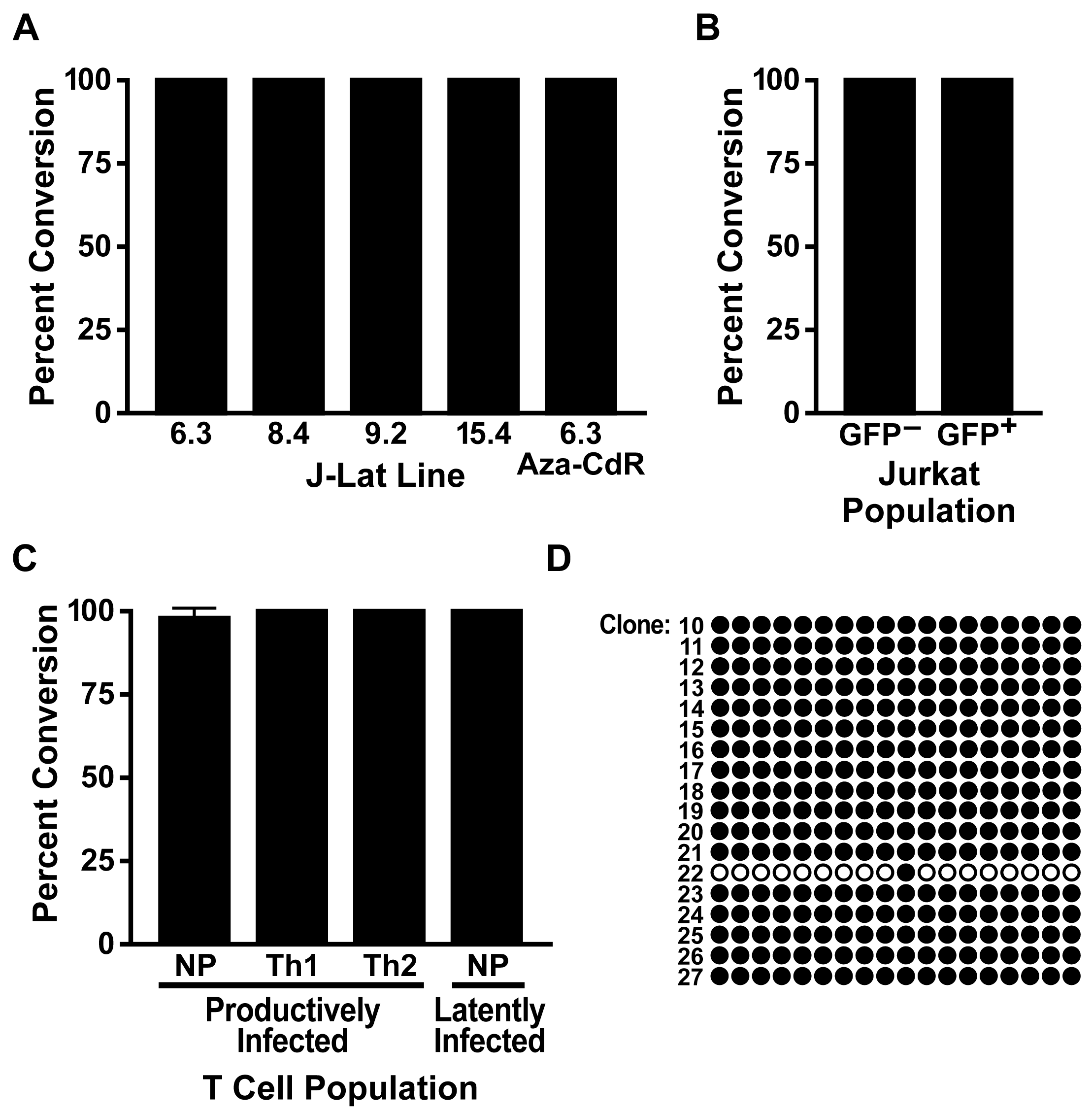

Supplement: Figure S5 — Sodium bisulfite conversion is highly efficient Mean percentage of cytosine-to-thymine conversion in non-CpG dinucleotides by sodium bisulfite treatment of (A) J-Lat cells, (B) Jurkat cells infected with HIV-1 R7/E−/GFP clone, or (C) CD4+ T cells. Error bars indicate standard deviation. (D) Results of bisulfite-mediated methylcytosine mapping of HIV-1 CpG island 2 for additional clones of latently infected CD4+ T cells stimulated under non-polarizing conditions. (0.62 MB TIF) [file ppat.1000495.s005.tif]

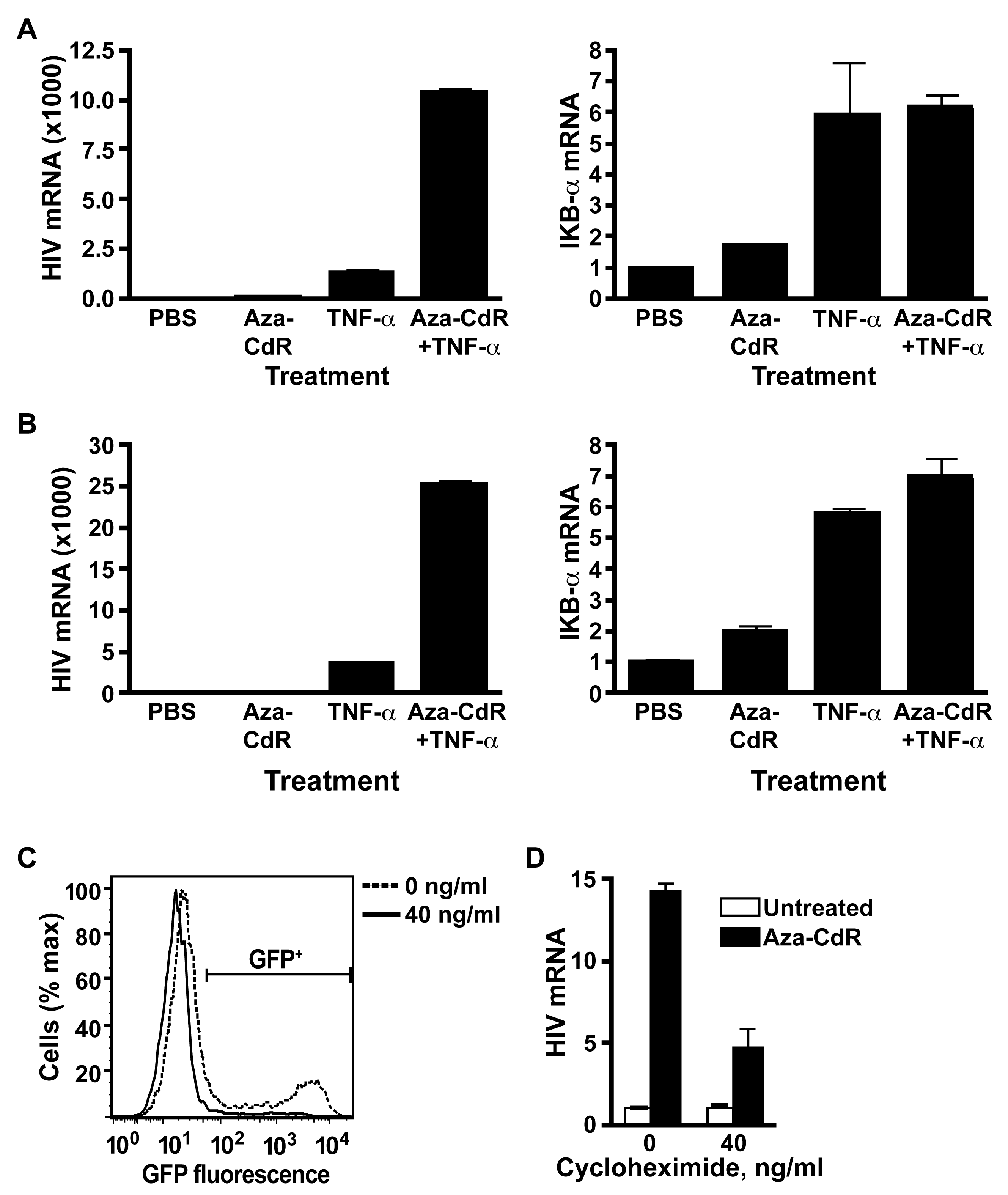

Supplement: Figure S6 — Synergistic activation of transcription is specific for the HIV-1 promoter. Steady-state mRNA levels were measured in (A) J-Lat 8.4 or (B) J-Lat 9.2. Quantity of HIV-1 (left panel) and IKB-α (right panel) mRNA was determined by reverse transcription and quantitative PCR after indicated treatments. Values are normalized to the PBS control. Error bars indicate standard deviation of quantitative PCR results. (C) Flow cytometric analysis of GFP expression in J-Lat cells after treatment with TNF-α, either in the presence or absence of cycloheximide. Histograms indicate GFP fluorescence. Gates indicate GFP-positive cells. (D) Levels of or HIV-1 (right panel) mRNA were determined by reverse transcription and quantitative PCR and normalized to cyclophilin mRNA. Cells were treated with aza-CdR, either in the presence or absence of cycloheximide. Error bars indicate standard deviation of qPCR results. (0.79 MB TIF) [file ppat.1000495.s006.tif]

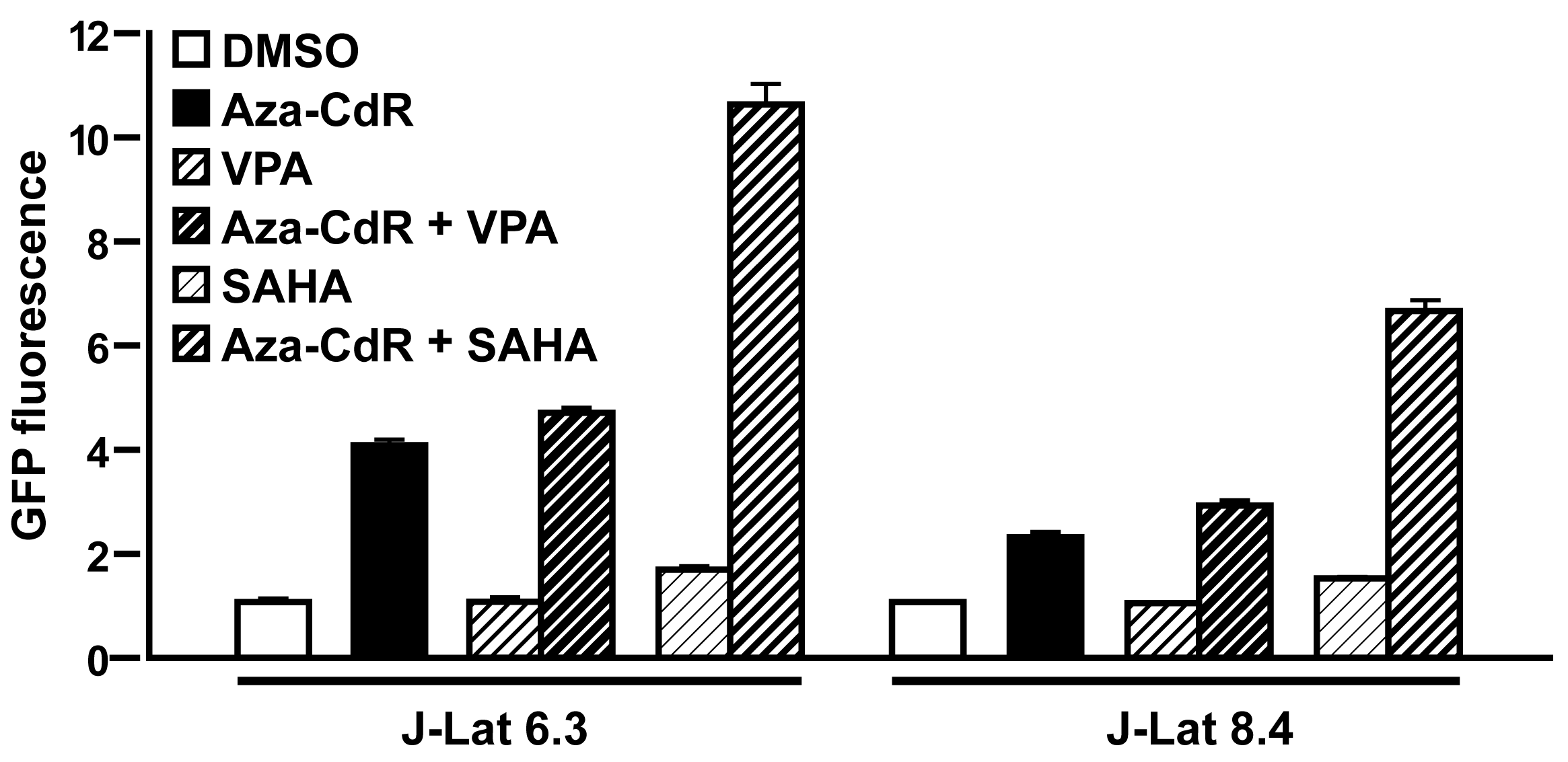

Supplement: Figure S7 — Aza-CdR and HDAC inhibitors do not synergistically reactivate latent HIV-1 Latent HIV-1 reactivation in the indicated J-Lat cell lines treated with aza-CdR, VPA, aza-CdR plus VPA, SAHA, or aza-CdR plus SAHA. GFP fluorescence was measured by flow cytometry and normalized to control cells treated with DMSO. Experiments were performed in triplicate and error bars indicate standard deviation. (0.32 MB TIF) [file ppat.1000495.s007.tif]

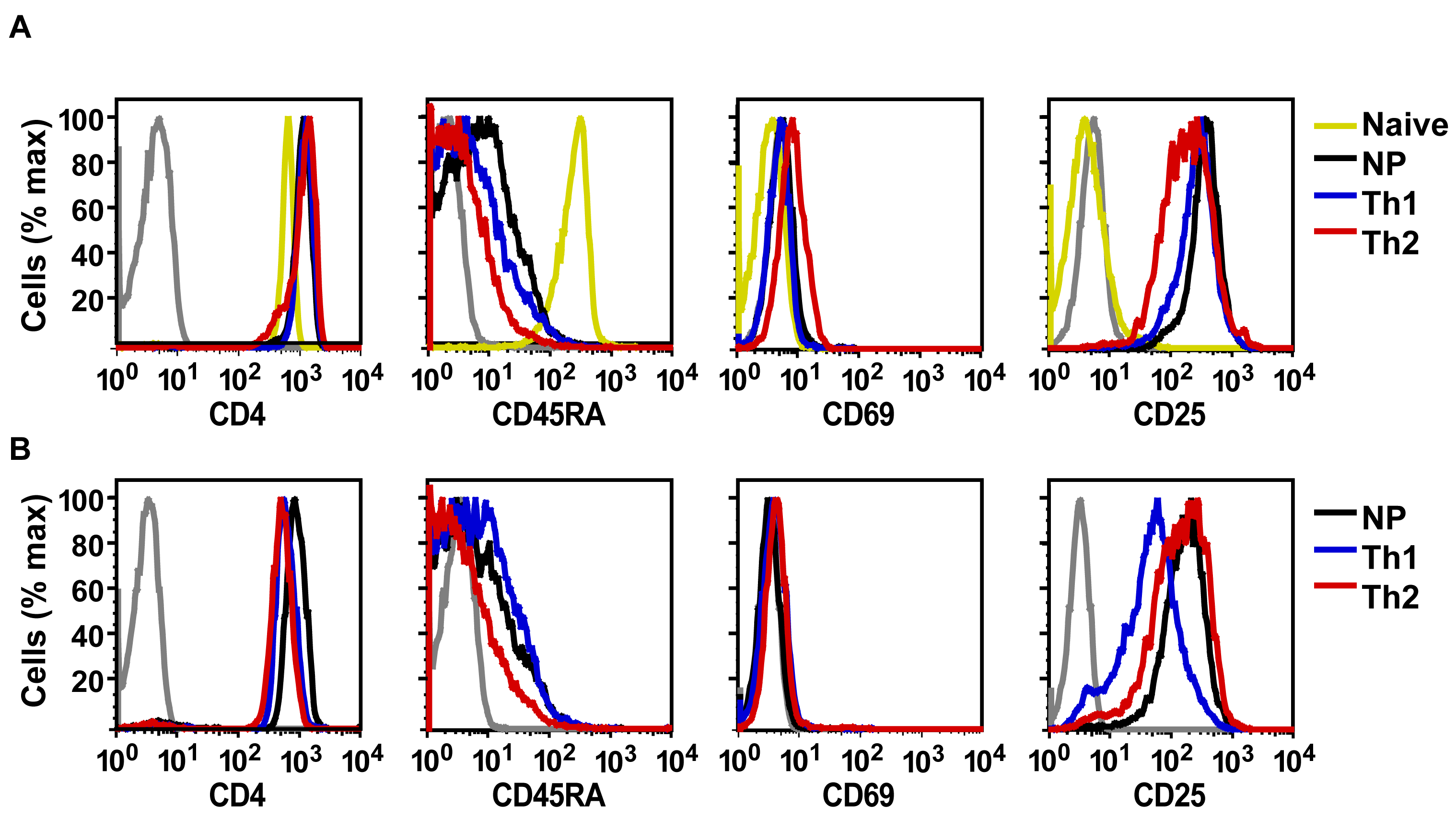

Supplement: Figure S8 — Phenotypic analysis of CD4+ T cells. Flow cytometric analysis of two donors, (A) and (B), is shown. Expression of CD4 (helper T cell), CD45RA (naïve T cell), CD69 (early activation), and CD25 (late activation) markers was determined after isolation and stimulation of naïve T cells. Grey histogram represents cells incubated with a non-fluorescent isotype control antibody. Data are representative of those from five different donors. (0.72 MB TIF) [file ppat.1000495.s008.tif]
