# Supplementary material for: Epigenetic Regulation of HIV-1 Latency by Cytosine Methylation
Source: PLoS Pathog. 2009 Jun 26;5(6):e1000495. doi: 10.1371/journal.ppat.1000495 (PMC2695767; doi:10.1371/journal.ppat.1000495)
Supplement: Table S1 — Reactivation of latent HIV-1. (0.04 MB DOC) [file ppat.1000495.s009.doc]

# Supplementary Table 1. Reactivation of latent HIV-1

|  | MFI, fold greater than PBS control | | |  |
| --- | --- | --- | --- | --- |
| J-Lat cell line | Aza-CdR | TNF-α | Aza-CdR+  TNF-α | Fold greater than additive effect^a^ |
|  |  |  |  |  |
| 6.3 | 1.8 | 7.1 | 196.4 | 21.9 |
| 8.4 | 1.6 | 2.5 | 101.9 | 24.7 |
| 9.2 | 1.1 | 2.3 | 76.1 | 22.5 |
| 15.4 | 1 | 1.7 | 47.1 | 17.7 |

^a^The magnitude of HIV-1 reactivation after treatment with both Aza-CdR and TNF-α was divided by the summed individual magnitudes after Aza-CdR or TNF-α treatment.
